# An anatomical and connectivity atlas of the tree shrew brain to bridge rodent and primate neuroanatomy

**DOI:** 10.1371/journal.pbio.3003773

**Published:** 2026-05-04

**Authors:** Xiaojia Zhu, Rui Bi, Haotian Yan, Qiyu Wang, Lin Li, Hongli Li, Long-Bao Lv, Cirong Liu, Yong-Gang Yao

**Affiliations:** 1 State Key Laboratory of Genetic Evolution & Animal Models, Yunnan Key Laboratory of Animal Models and Human Disease Mechanisms, Yunnan Engineering Center on Brain Disease Models, and KIZ-CUHK Joint Laboratory of Bioresources and Molecular Research in Common Diseases, Kunming Institute of Zoology, Chinese Academy of Sciences, Kunming, Yunnan, China; 2 Institute of Neuroscience, State Key Laboratory of Genetic Evolution and Animal Models, Center for Excellence in Brain Science and Intelligence Technology, Chinese Academy of Sciences, Shanghai, China; 3 Kunming College of Life Science, University of Chinese Academy of Sciences, Kunming, Yunnan, China; 4 National Research Facility for Phenotypic & Genetic Analysis of Model Animals (Primate Facility), National Resource Center for Non-Human Primates, Kunming Institute of Zoology, Chinese Academy of Sciences, Kunming, Yunnan, China; Oxford University, UNITED KINGDOM OF GREAT BRITAIN AND NORTHERN IRELAND

## Abstract

The tree shrew (*Tupaia belangeri*), phylogenetically proximal to primates, serves as a critical model for evolutionary neurobiology and disease mechanisms. High-resolution MRI provides a unique opportunity to refine its neuroanatomical architecture and facilitate cross-species comparisons. Here, we present a comprehensive, ultra-high-resolution (9.4T) MRI atlas of the tree shrew brain, integrating structural and diffusion imaging to resolve fine-scale anatomical features and whole-brain connectivity gradients. Our comparative analysis characterizes the tree shrew as a distinct evolutionary mosaic: the cerebellum exhibits pronounced volumetric expansion and connectivity gradients recapitulating those of primates, whereas the hippocampus retains rodent-like architectural scaling yet preserves evolutionarily conserved longitudinal functional axes. Moving beyond these regional adaptations, we uncovered a universal organizational principle: geometry–gradient coupling (GGC)—the fundamental constraint of brain shape on functional organization. By systematically linking geometric eigenmodes to connectivity gradients across diverse species (from mice to humans), we demonstrate that despite dramatic morphological divergence, the spatial alignment between brain geometry and functional organization remains evolutionarily invariant. Collectively, these results establish the tree shrew as a pivotal phylogenetic bridge and provide a neuroanatomical benchmark for deciphering the interplay between structural diversity and universal biophysical constraints.

## Abstract 2

The tree shrew is a key evolutionary bridge between primates and other mammals. This study presents a comprehensive, anatomical and connectivity atlas of the tree shrew brain, revealing that the spatial alignment between brain geometry and functional organization remains evolutionarily invariant.

## Introduction

The tree shrew (*Tupaia belangeri*), a small arboreal mammal phylogenetically close to primates, has served as a unique model for both disease modeling and neurobiology [1–4]. Several recent studies utilizing the tree shrew have provided profound insights into the mechanisms of neuropsychiatric disorders [2,5–8] and fundamental neural hierarchies [9]. Consequently, characterizing the fine-scale wiring architecture and connectomes of the tree shrew brain is essential for modeling brain diseases and for bridging the comparative evolutionary gap between rodents and primates.

Magnetic resonance imaging (MRI) provides a vital tool for mapping brain anatomy and connectivity [10]. While high-resolution MRI has successfully delineated fine-grained anatomical features in primates [11–13] and rodents [14,15], current investigations of the tree shrew brain [16–19] remain constrained by limited resolution (120–300 μm^3^ versus primate benchmarks of 10–50 μm^3^) [11–13]. This resolution gap has obscured functionally critical substructures, such as cerebellar folia (<100 μm thickness) and hippocampal subfield cytoarchitecture, preventing a definitive determination of whether these regions exhibit primate-like specialization.

Beyond discrete anatomical boundaries, defining a comprehensive atlas requires capturing the continuous topography of functional organization. Conceptually, these patterns are described as “gradients”—continuous spatial axes that characterize the gradual transitions in connectivity profiles underlying functional organization. Crucially, these gradients provide a framework for integrating multi-scale organizational principles, linking macroscale functional hierarchies to underlying patterns of connectivity [20]. To quantify this organization, analytical approaches such as diffusion map embedding [20] have been employed to project high-dimensional connectivity data into lower-dimensional manifolds [21,22]. Recent analyses have successfully mapped these hierarchical axes, revealing detailed connectivity gradients in cortical [20,23], cerebellar [24–27], and hippocampal [28–31] structures across mammals. However, the manifestation of these connectivity gradients manifest in the tree shrew remains uncharacterized, hindering the characterization of species-specific neural specializations and evolutionary adaptations in brain organization.

Furthermore, emerging theoretical frameworks suggest that these gradients are not arbitrary but are physically constrained by the brain’s shape—its geometry. This hypothesis, often analyzed through geometric eigenmodes, posits a tight structure-function coupling where the physical boundaries of neural tissue constrain the propagation of functional activity [32], manifesting as a high spatial similarity between brain geometry and functional gradients (FGs). While this geometry–gradient coupling (GGC) has been demonstrated in humans, whether it represents an evolutionarily conserved principle in the tree shrew remains to be determined.

In this study, we present a comprehensive anatomical and connectivity atlas of the tree shrew brain derived from ultra-high-resolution *ex vivo* MRI (9.4T). We constructed a population-averaged template with fine-grained whole-brain parcellation to reveal refined anatomical architectures. Leveraging multi-shell diffusion MRI data, we characterized the principal structural connectivity gradients across the cerebral cortex, cerebellum, and hippocampus. Crucially, we evaluated structure-function coupling by systematically linking brain geometry (eigenmodes) to connectivity gradients across five species (mouse, tree shrew, marmoset, macaque, and human). This whole-brain framework establishes an essential resource for cross-species evolutionary neuroanatomy and translational brain disease modeling.

## Results

### Construction of the tree shrew brain population template

**Fig 1:**
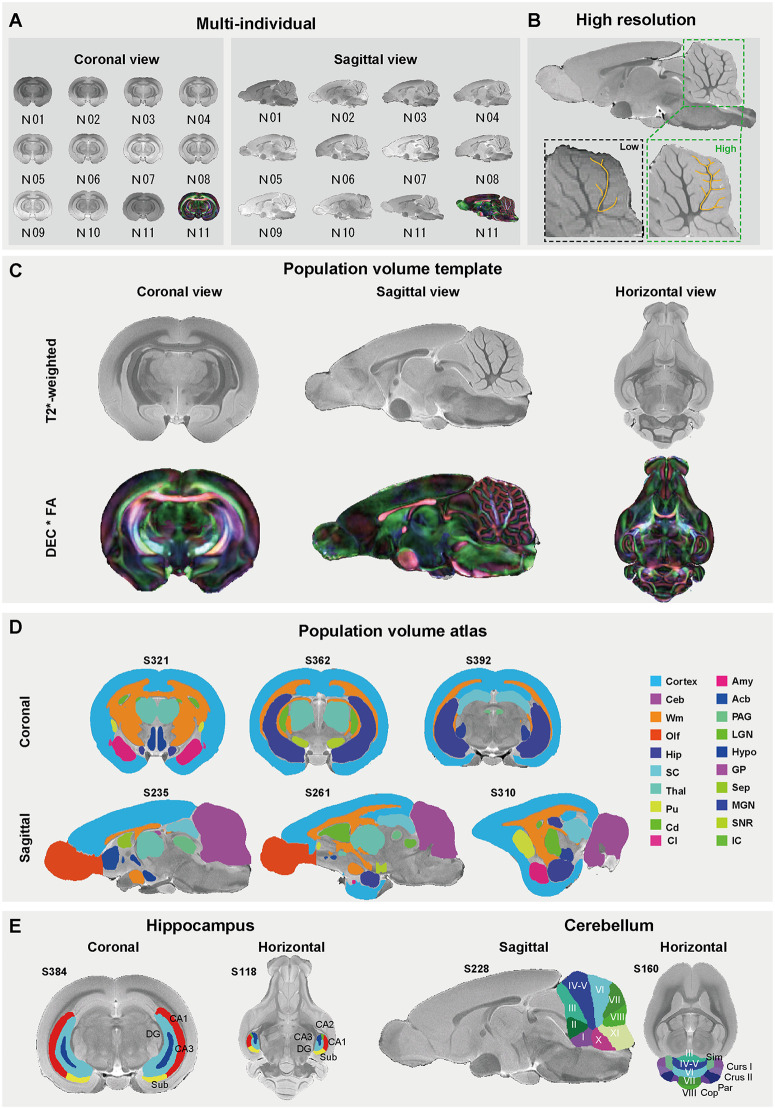
Population template and volume-based brain parcellation atlas of the tree shrew brain. **(A)** Multi-subject coronal (*left*) and sagittal (*right*) views of T2*-weighted MRI and FA-weighted DEC images. DEC, directionally encoded color; FA, fractional anisotropy. Female, N08 and N10; the remaining individuals are male. **(B)** Ultra-high-resolution T2*-weighted MRI (green box: 50 × 50 × 75 μm^3^) demonstrating fine-scale cerebellar lobular architecture. This contrasted to conventional-resolution data (black box: 100 × 100 × 200 μm^3^) with missing structural granularity. **(C)** Population-averaged volume templates of the tree shrew brain of multi-individual T2*-weighted MRI (*top*) and FA-weighted DEC (DEC*FA, *bottom*), displaying in triplanar views (coronal, sagittal, and horizontal). **(D)** Whole-brain parcellation in coronal (*top*) and sagittal (*bottom*) views, with slice positions numerically labeled. Anatomical regions are color-coded and abbreviated as follows: cerebral cortex (Cortex), cerebral white matter (Wm), amygdala (Amy), thalamus (Thal), medial geniculate nucleus (MGN), lateral geniculate nucleus (LGN), caudate (Cd), putamen (Pu), nucleus accumbens (Acb), claustrum/endopiriform claustrum (Cl), hypothalamus (Hypo), septum (Sep), globus pallidus (GP), inferior colliculus (IC), superior colliculus (SC), periaqueductal gray (PAG), substantia nigra (SNR), hippocampus (Hip), cerebellum (Ceb), and olfactory bulb (Olf). **(E)** Subregional parcellation of the hippocampus (*left*) and cerebellum (*right*), with slice positions indicated above each panel.

We acquired high-resolution *ex vivo* brain images of 11 adult tree shrews (9 males and 2 females) using a 9.4T MRI scanner (Fig 1A). Structural imaging employed a T2*-weighted sequence with ultra-high resolution (50 × 50 × 75 µm^3^). Diffusion MRI data were acquired from one individual (N11) with 125 µm isotropic resolution and multi-shell sampling (*b*-values = 50, 1,500, 3,000 s/mm^2^ for 6, 30, and 60 directions, respectively). This high-resolution data enabled clear discrimination of cerebellar folia and lobules, as demonstrated by the cerebellar white matter branching patterns (Fig 1B). Crucially, such detailed patterns were indiscernible in conventional low-resolution imaging (100 × 100 × 200 μm^3^) [16] from the same individual (Fig 1B).

To establish a standardized reference for cross-individual and cross-study comparisons, we generated a population-averaged brain template (Fig 1C) from the high-resolution structural MRI data using ANTs [33]. We utilized a multi-stage iterative approach with normalized mutual information as the similarity metric for template construction, initializing with the highest quality individual dataset (N11). Diffusion MRI datasets were co-registered to this template using a two-step affine and symmetric diffeomorphic registration [33]. The fidelity of the diffusion tensor orientations was manually inspected by visualizing the primary eigenvector (V1) maps in the template space to ensure consistent directional alignment (S1A Fig). This dataset formed the foundation for all subsequent anatomical and connectional analyses. To promote data sharing, we developed a web-based platform for interactive 3D exploration of the tree shrew brain atlas (http://www.treeshrewdb.org/MRI/) (S1B Fig).

### Fine-grained anatomical architecture of the tree shrew brain

We developed a whole-brain atlas based on the population template described above. This atlas spanned 20 macroscale regions (including cerebral cortex, thalamus, cerebellum, hippocampus) (Fig 1D), segmented in alignment with the stereotaxic coordinates and anatomical delineations provided by Zhou and Ni [34]. Given their pivotal roles in arboreal adaptation, where demands for millisecond motor precision (cerebellum) and three-dimensional spatial mapping (hippocampus) confer survival advantages [35–37], we further delineated 16 cerebellar lobules and 5 hippocampal subfields at higher resolution (Fig 1E). We validated the cytoarchitectonic boundaries through consensus-based cerebellar fissures (S1C Fig), while hippocampal subfield segmentation adhered to cytoarchitectural discontinuities (S1D Fig). This parcellation atlas enabled precise mapping of fine-scale neuroanatomical features unique to the tree shrew, facilitating quantitative assessment of fine-grained subregional brain volumes and cross-species morphological comparisons. Using this population atlas, we were able to calculate the volume of the whole brain and each macroscale region (S1E Fig and S1 Table). The tree shrew brain volume was approximately 3,252 mm^3^, with the cortex being the largest region (about 1,063 mm^3^, ~33% of total volume), followed by the cerebellum (435 mm^3^, ~13.4%), cerebral white matter, olfactory bulb, and hippocampus. Among the 16 cerebellar subregions (S1F Fig and S2 Table), lobules I and II were the smallest, while lobules IV–V and the paraflocculus (PFL)—regions implicated in limb/trunk motor coordination and oculomotor control/vestibulo-ocular reflexes, respectively [38–40]—were the largest. Among hippocampal subregions (S1G Fig and S3 Table), the dentate gyrus (DG) was the largest (28.7%), while CA2 was the smallest.

To assess cross-individual consistency, we registered the template-based parcellation to individual brains and quantified the regional volumes (S2A Fig). While absolute volumes varied substantially across individuals (S2B Fig), the relative volumetric distributions remained highly stable. Overall, most regions/subregions exhibited a small coefficient of variation (CV < 0.1; S2C–S2E Fig). Notably, cerebellar lobule X showed an elevated variability (CV = 0.12), whereas all the hippocampal subregions (CA1, CA2, CA3, DG, and Sub) displayed minimal variance (0.02–0.07) among all 11 individuals under study. These findings indicate that despite substantial inter-individual differences in absolute size, the relative topographical organization remains conserved. Furthermore, a supplemental exploration of sex differences indicated that neuroanatomical organization was broadly comparable between sexes. While total brain volume remained consistent, we observed only subtle variations, with females exhibiting a slight trend toward higher absolute and relative volumes in specific subcortical regions (notably Acb and SC) and a marginally lower proportion of cortical volume (S3A and S3B Fig). Therefore, the population-averaged template established here provides a reliable reference for disentangling species-specific brain structural features.

### High-resolution surface morphometry of the tree shrew brain

**Fig 2:**
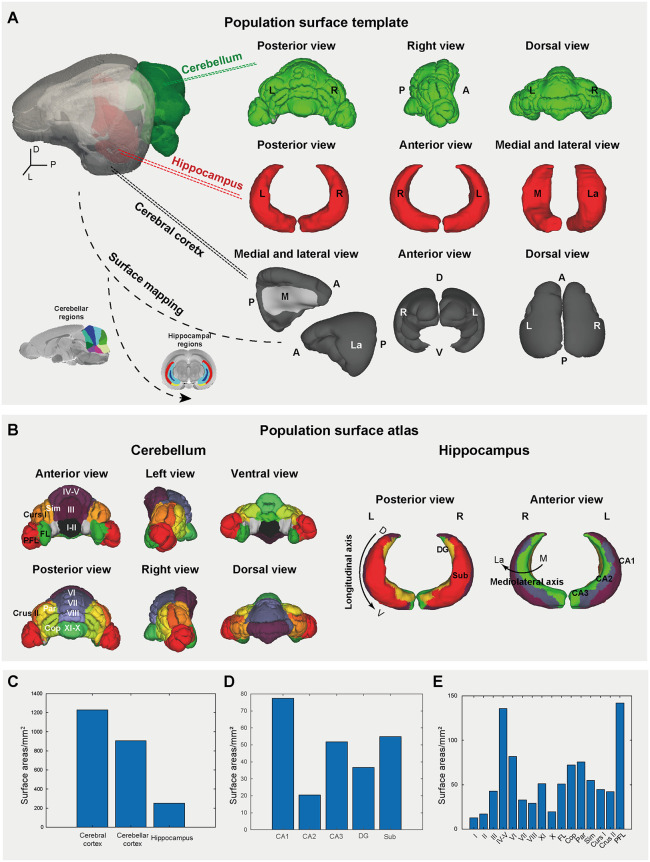
Surface-based brain parcellation atlas of the tree shrew. **(A)** Surface templates for the cerebellum (*green*), hippocampus (*red*), and cerebral cortex (*gray*). Anatomical orientations are labeled as left (L), right (R), anterior (A), posterior (P), medial (M), lateral (La), dorsal (D), and ventral (V). **(B)** Surface-based parcellation of the cerebellum (*left*) and hippocampus (*right*), generated by projecting volume-derived partitions onto their respective morphometric surfaces. **(C–E)** Surface area distributions across broad anatomical regions (C), hippocampal subregions (D), and cerebellar lobules (E). The data underlying this Figure can be found in S1 Data.

Surface representations in 3D provide the essential computational substrate for a detailed geometric analysis of complex brain structures. To enable 3D geometric characterization and surface-based morphometry of fine-scale neuroanatomy, we used the structural MRI to reconstruct surface meshes of the cerebral cortex, hippocampus, and cerebellar cortex using our previously optimized animal-adapted surface reconstruction pipeline [25,41] based on FreeSurfer [42]. We generated topologically optimized, anatomically precise meshes for bilateral cerebral cortices, hippocampi, and the entire cerebellum, providing a geometrically faithful representation of gray/white matter interfaces (Fig 2A). By mapping the volumetric parcellations to the cortical, hippocampal, and cerebellar surfaces, we generated surface-based partitions (Fig 2B), facilitating systematic quantification of surface areas ranging from broad anatomical structures to fine subregions (Fig 2C–2E and S4 Table). The surface area of the cerebral cortex was approximately 1229.48 mm^2^, followed by the cerebellum (905.10 mm^2^), and the total surface area of the bilateral hippocampus was 250.10 mm^2^ (Fig 2C). Among the 5 hippocampal subregions, the CA1 had the largest surface area, followed by Sub, CA3, DG, and CA2 (Fig 2D). Among 16 cerebellar subregions (Fig 2E), lobules I and II had the smallest surface area, whereas lobules IV–V and PFL were the largest, a pattern closely mirroring volumetric measurements. Collectively, these surface-based frameworks facilitate multiscale morphometry, bridging macro anatomical profiling (e.g., cortical expansion) and microstructural complexity (e.g., cerebellar foliation), while providing a unified geometric scaffold for size-independent cross-species comparisons.

### Cross-species brain structure comparison showing the unique features of the tree shrew

**Fig 3:**
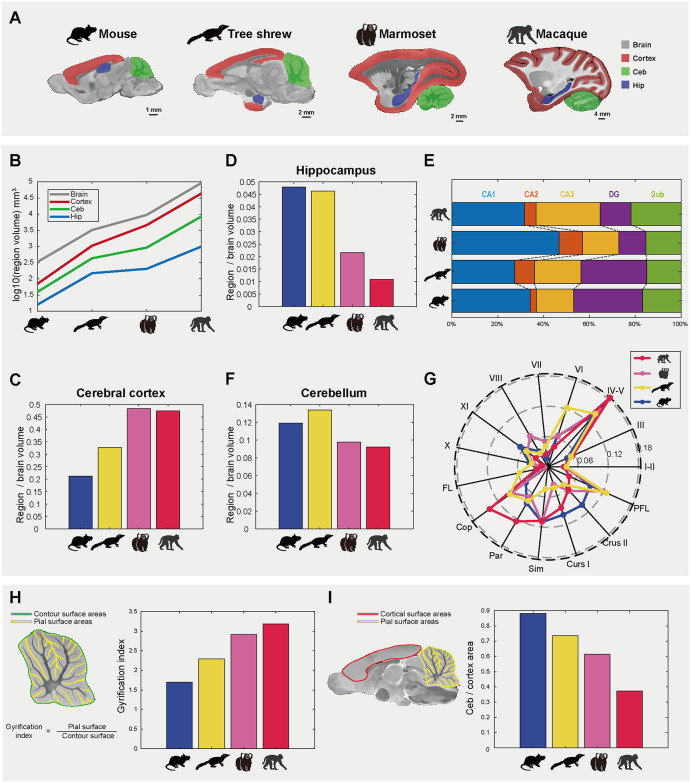
Cross-species comparisons based on brain parcellation. **(A)** Parcellation maps for brain, cortex, cerebellum (Ceb), and hippocampus (Hip) in the mouse, tree shrew, marmoset, and macaque. Each map has a scale bar reflecting species-specific brain dimensions. **(B)** Absolute volumes of brain, cortex, cerebellum (Ceb), and hippocampus (Hip) across the mouse, tree shrew, marmoset, and macaque. **(C, D)** Relative volumes (normalized to total brain volume) of cerebral cortex **(C)** and hippocampus **(D)** across species. **(E)** Relative volumes for the hippocampal subregions (normalized to hippocampal volume) across species. **(F)** Relative volumes (normalized to total brain volume) of cerebellum across species. **(G)** Relative volumes for the cerebellar lobules (normalized to cerebellar volume) across species. The abbreviations are defined in the Materials and Methods section. **(H)** Cerebellar gyrification index (GI) across species. GI quantifies folding complexity of the cerebellar cortex, calculated as the ratio of the pial surface area (yellow line) to the outer contour surface area (green line). Higher GI values indicate greater cortical folding. **(I)** Cerebellar-to-neocortical surface area ratio across species. Ratio was calculated as cerebellar pial surface area (Ceb; yellow line) divided by neocortical surface area (cortex; red line). The data underlying this Figure can be found in S1 Data.

We conducted cross-species brain comparisons using 3 common experimental animals (macaque, *Macaca mulatta*; marmoset, *Callithrix jacchus*; mouse, *Mus musculus*), with reference to the tree shrew, to characterize structural similarity and evolutionary development. Utilizing widely used templates [12,41,43,44] and atlases [45–47] (Fig 3A), we found that absolute volumes of the whole brain and three major regions generally followed phylogenetic status (mouse < tree shrew < marmoset < macaque), with cortical volume showing the most linear increase (Figs 3B and S4A). Using normative species body weights, we quantified absolute brain volume-to-body mass ratios (S4B Fig). This revealed the tree shrew exhibited the highest ratio among the studied species—mildly exceeding the marmoset and substantially surpassing the mouse and macaque. This exceptional brain-to-body ratio in the tree shrew, which significantly surpasses that of rodents and closely approaches the proportions seen in primates (marmoset), is consistent with previous anatomical findings [48]. We calculated the relative volumetric distributions (normalized to total brain volume) to show potential species-specific adaptations among these species, which could not be demonstrated by the absolute volume metrics. We observed a stepwise increase in relative cortical volume (mouse < tree shrew < marmoset < macaque) (Figs 3C and S4A), indicating substantial neocortical expansion in the tree shrew relative to mice, albeit less pronounced than in primates.

The relative hippocampal volume of the tree shrew was comparable to the mouse but higher than in non-human primates (Figs 3D and S4A). Direct comparison of hippocampal subregions showed that the tree shrew shared higher similarity with the mouse (Figs 3E and S4A), suggesting the retention of a rodent-like subregional scaling pattern. Conversely, the tree shrew exhibited the largest relative cerebellar volume, surpassing all other species (Figs 3F and S4A). This marked cerebellar enlargement mirrors the pattern of cortical dominance in primates. The high locomotion of the tree shrew relative to primates and the mouse [35,49] could be a potential reason why the tree shrew has an expanded cerebellum. We further compared inter-species cerebellar parcellation proportions to identify the cerebellar lobules driving this volumetric expansion (Fig 3G). We found that the tree shrew exhibited a disproportionately large proportion of lobule VI (linked to motor control and spatial navigation [50]), which is double to triple the values of lobule VI in the macaque, marmoset, and mouse. The FL lobule (linked to eye movements [51,52]) was also expanded in the tree shrew as compared to the other species. The relative volume of the Sim lobule (linked to cognitive [53]) in the tree shrew (4.4%) was remarkably smaller than those of non-human primates and the mouse (~11%) (Fig 3G). Using the proportional contributions of cerebellar lobules to total cerebellum volume, we performed Pearson’s correlation analysis to quantify volumetric similarity across species. This analysis revealed a stronger volumetric similarity of the tree shrew parcellation patterns to non-human primates (marmoset, *r* = 0.68; macaque, *r* = 0.53) than to mouse (*r* = 0.50). These findings indicated that the overall cerebellar parcellation volume distribution in the tree shrew was slightly closer to primates than to mouse.

We computed the cerebellar gyrification index (GI), which quantifies folding complexity by calculating the ratio of the external contour surface area to the total pial surface area [54,55]. Higher GI values would reflect an increased gyral-sulcal elaboration and suggested advanced neuroanatomical organization that was associated with enhanced intelligence scores [56]. We generated high-fidelity reconstructions of both the inner (pial) and outer (superficial contour) surface areas to calculate the GIs of the cerebellar cortex across the four species. The GI values of the cerebellar cortex showed a progressive increase from the mouse to the macaque, with the lowest value in the mouse (1.7), followed by the tree shrew (2.3), marmoset (2.9), and macaque (3.2) (Fig 3H), reflecting an evolutionary trajectory of increasing folial complexity. Finally, we quantified the cerebellar-to-neocortical surface area ratio (Fig 3I), observing a pattern inverse to GI: mouse (88.2%) > tree shrew (73.6%) > marmoset (61.5%) > macaque (37.3%). While human ratios (~80%) suggest a link between relative cerebellar expansion and cognition [57], the non-linear pattern observed here implies a complex evolutionary trajectory rather than a simple progression across mammals.

Taken together, the tree shrew displays a distinctive mosaic of neuroanatomical features: a hippocampus preserving rodent-like scaling, a cortex transitional between orders, and a cerebellum exhibiting unique, pronounced expansion.

### The tree shrew and primates have similar cerebellar structural connectivity gradients

**Fig 4:**
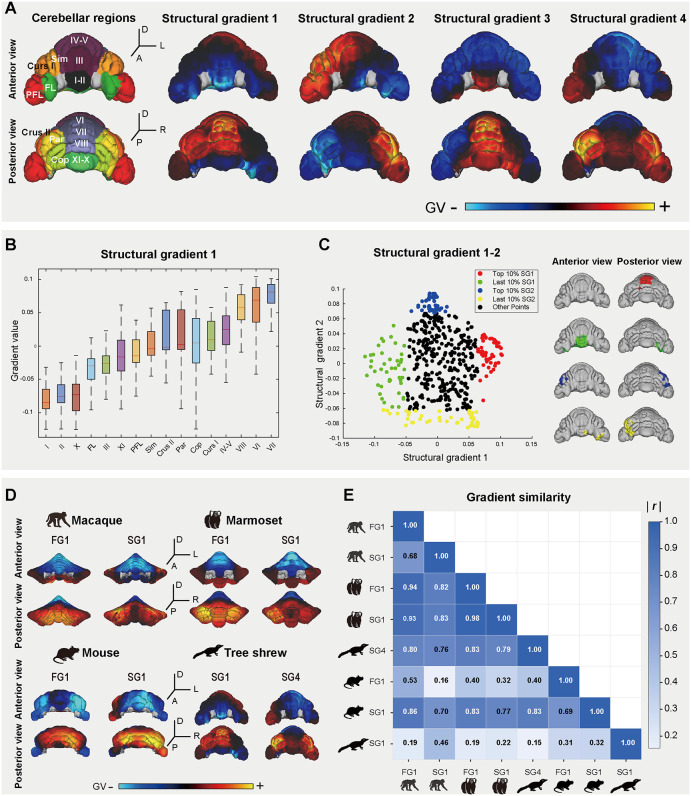
Structural connectivity gradients of the tree shrew cerebellum. **(A)** Surface-based cerebellar atlas (Cerebellar regions; *left*) and structural gradients 1–4 (SG1–SG4). Gradient values (GVs) are color-mapped. **(B)** SG1 values across cerebellar lobules, displayed as a box plot (median ± interquartile range). **(C)** Spatial localization of extremal gradient values (top/last 10%) for SG1 and SG2, overlaid on the surface of cerebellar cortex. **(D)** Surface-based gradient mappings in cerebellums: FG1/SG1 in the macaque, marmoset, and mouse; SG1/SG4 in the tree shrew. **(E)** Absolute Pearson coefficient of correlation (| *r |*) comparison among the structural and functional gradients across species based on the regional-averaged-GV with hemispheric differentiation. The data underlying this Figure can be found in S1 Data.

To characterize the macro-scale organizational principles of the tree shrew, we applied diffusion map embedding [20] to structural connectivity matrices derived from the diffusion MRI data of a single high-resolution individual (N11), generating connectivity gradients across cerebellar, hippocampal, and cerebral cortical regions. We focused on the first four dominant gradients generated through this process, which captured the major variance and represented principal organizational axes within different brain regions [20]. Given the well-documented FG along the anteroposterior (AP) axis in primates and rodents [24–27], we first examined whether the tree shrew cerebellum exhibits a similar architecture. In the tree shrew, the first structural connectivity gradient (SG1) of the cerebellum showed the largest amount of variance (10.4%). SG1 exhibited an overall AP axis spanning lobules I–II to VI–VIII, peaking at lobule VI as indicated by its maximal gradient value (GV) (Figs 4A and S5A). Notably, this gradient extremum coincides with the significant volumetric expansion of lobule VI (Fig 3G), linking structural connectivity to anatomical specialization. SG2 (9.7% of variance) demonstrated a left-right distribution across cerebellar hemispheres, traversing the vermis to connect contralateral regions (Figs 4A and S5B). This pattern mirrored the FGs observed in humans [24,27] (FG3 and FG4) and the marmoset [25] (FG3), suggesting an evolutionary conservation of cerebellar laterality [24,58]. The SG3 (9.2% of variance) exhibited the highest GV predominantly to the vermis (lobules I–II and VI–X) and the lowest GV to the Cop and PFL. The GVs of SG3 in most of the hemispheric regions were rather low, with the lowest value in Crus I and the Sim (Figs 4A and S5C). The overall pattern of SG3 mirrored the FG4 as observed in the marmoset [25]. Crucially, SG4 (8.4% variance) progressed from primary motor areas (lobules I–VI) to peak in non-motor regions (Crus I–II, Cop, Par) (Figs 4A and S5D), prominently recapitulating the motor/non-motor organizational axis observed in primates [25,26].

We calculated the cerebellar structural gradients (SGs) in the macaque, marmoset, and mouse, and integrated our previously published functional gradients (FGs) [25,26] to quantitatively evaluate the conservation of cerebellar gradients across these species (Figs 4D and S6A–S6F). Comparison of averaged gradients within conserved partitions revealed three key insights: First, we found the SGs and FGs exhibited a high degree of similarity in the macaque, marmoset, and mouse (Figs 4D, 4E, and S6A–S6F). This result demonstrated a strong structure-function coupling in the cerebellum. Second, cerebellar gradient 1 (both SG1 and FG1) exhibited a distinct AP axis in all four species, indicating a conserved AP differentiation in the cerebellum. Third, and most significantly, the tree shrew SG4 exhibited a stronger similarity to the primate primary gradient (SG1/FG1) than the tree shrew’s own SG1 did (Fig 4E). Moreover, this SG4–FG1 correlation was significantly stronger in primates than in mice. Given that SG4 explains comparable variance (8.4%) to SG1 (10.4%), these results suggest that tree shrew SG4 represents an evolutionarily conserved functional axis aligning with the primate FG1. The strong coupling of these gradients with their primate homologs suggests that the tree shrew has evolved cerebellar connectional architectures that resemble those of primates.

### Hippocampal structural connectivity gradients of the tree shrew follow an evolutionary conserved longitudinal organization

**Fig 5:**
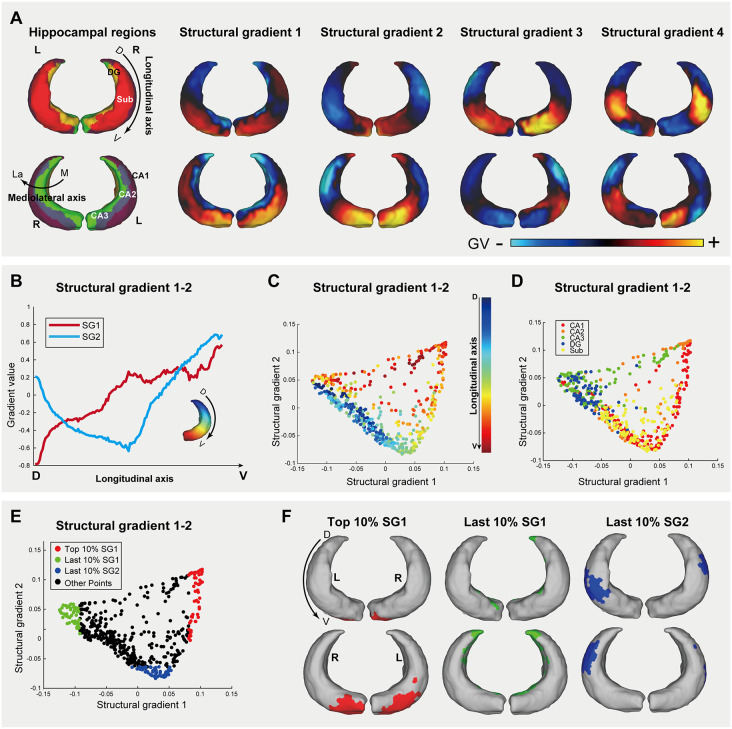
Structural connectivity gradients of the tree shrew hippocampus. **(A)** Bilateral hippocampal surface atlas (hippocampal regions) and structural gradients 1–4 (SG1–SG4). Arrows denote longitudinal axis orientation (dorsoventral axis, DV) or short axis (medial to lateral, M-La), as indicated. Abbreviations: L, left; R, right; M, medial; La, lateral; D, dorsal; V, ventral. **(B–F)** Left hippocampus analyses. (B) Spatial alignment of SG1 and SG2 values along the longitudinal axis (DV-axis). (C) DV-axis progression of SG1 and SG2 values, color-mapped by DV-axis position. (D) Subregional heterogeneity of SG1 and SG2 across hippocampal subregions (color-coded). (E) Spatial localization of extremal gradient values (top/last 10%) for SG1 and SG2. (F) Spatial localization of extremal gradient values for SG1(top/last 10%) and SG2 (last 10%), overlaid on the surface in the left hippocampus. Results for right hippocampus of the tree shrew are presented in S7 Fig. The data underlying this Figure can be found in S1 Data.

Having established the primate-like organization of cerebellar gradients, we next examined the hippocampus. Our analysis revealed that the principal connectivity gradient aligns robustly with the longitudinal axis (dorsal-to-ventral, DV), a topographical organization that captures both continuous transitions and discrete functional domains (Fig 5A). The SG1 (16% of variance) demonstrated a longitudinal axis differentiation, showing gradual attenuation from the dorsal to the ventral poles (Fig 5A and 5B). The SG2 (13.7% of variance) displayed a distinct bimodal pattern, decreasing from the ventral pole to the body before rising again at the dorsal pole (Fig 5A and 5B). These primary gradients align with the longitudinal functional organization observed in primates [28–30] and mice [31], suggesting evolutionary conservation [59–61]. Notably, both gradients showed discrete modular organization rather than smooth transitions along the longitudinal axis (Fig 5A and 5B). This compartmentalization was particularly evident in SG3 (11.9 of variance) and SG4 (10.2% of variance), suggesting three distinct hippocampal segments along the longitudinal axis with bilateral symmetry (Fig 5A). We postulate that these domains arise from the interaction between the intrinsic longitudinal axis and a mediolateral hierarchy defined by subfield-specific cytoarchitecture [62].

To systematically map the spatial correspondence between connectivity gradients and hippocampal anatomy, we visualized the gradient manifold (SG1 versus SG2) annotated with longitudinal coordinates and subregional boundaries. Within this gradient space, longitudinal positions emerged as a spatially continuous trajectory (Fig 5C), confirming the robust alignment of the principal gradients with the longitudinal axis. In contrast, mapping hippocampal sub-regions revealed distinct clustering within the gradient space (Fig 5D), a pattern that mirrors the intrinsic mediolateral differentiation of the hippocampus [62,63]. Extreme value analysis further anchored these gradients to specific anatomical landmarks: the highest 10% of SG1 values localized to the ventral pole, while the lowest 10% mapped to the dorsal and medial regions (Fig 5E and 5F). This topography displayed robust bilateral symmetry, confirmed by independent replication in the right hemisphere (S7A–S7D Fig). Collectively, these findings demonstrate that tree shrew hippocampal gradients encode an evolutionarily conserved longitudinal axis while simultaneously capturing discrete subregional architecture.

### Conserved geometry–gradient coupling across multiple species

While our regional analyses of the cortical, cerebellar, and hippocampal morphology collectively delineate a distinct evolutionary mosaic, a fundamental question remains regarding the rules governing whole-brain organization. Recent theoretical frameworks in humans suggest that neural activity is not arbitrary but is tightly constrained by the brain’s geometry. Specifically, functional organization in human subcortical structures—including the hippocampus, hypothalamus, and striatum—was found to be predominantly governed by long-wavelength geometric eigenmodes, exhibiting near-perfect GGC (|*r*| ≥ 0.93) [32]. This suggests that the functional topography of these regions is derived directly from their physical shape. However, whether this GGC represents a universal biophysical law that transcends species-specific variations—from the lissencephalic mouse to the gyrified primate—remains to be determined. To test the evolutionary conservation of this principle, we systematically investigated GGC in the tree shrew and extended our validation to humans, macaques, marmosets, and mice.

**Fig 6:**
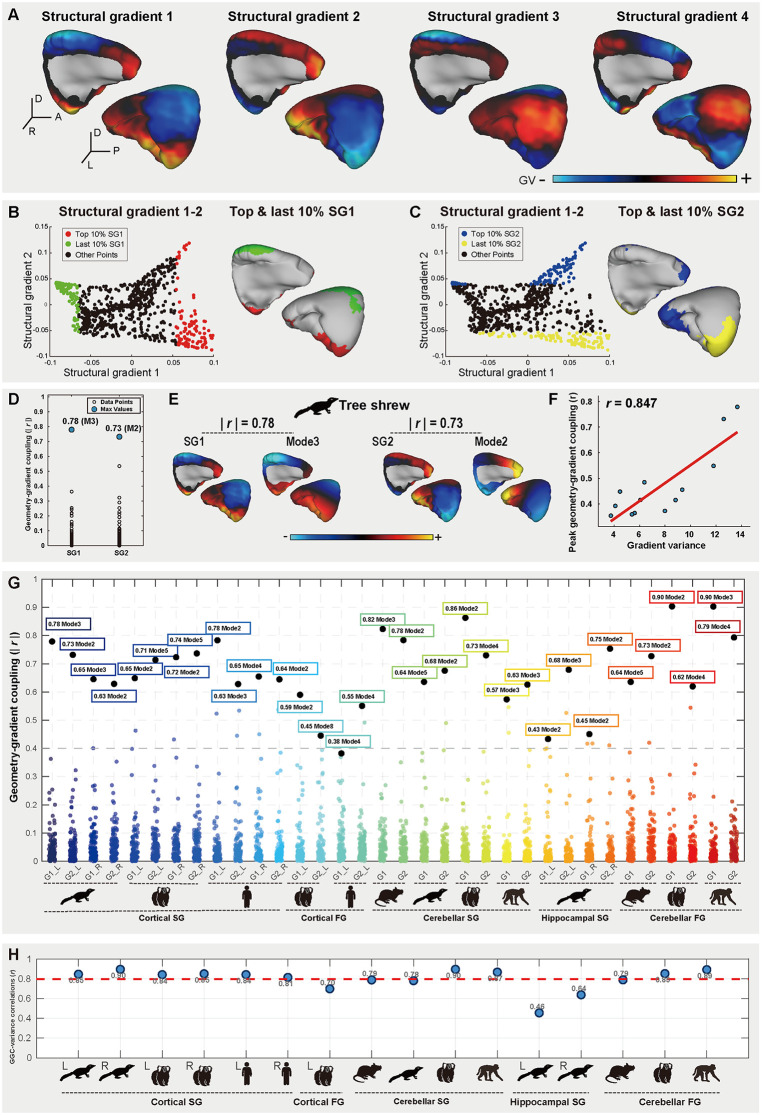
Structural connectivity gradients of the tree shrew left cerebral cortex and peak geometry–gradient coupling (GGC) across species. **(A)** Cortical surface maps of the first four structural gradients (SG1–SG4) in the left cerebral cortex, with color-encoded by gradient value (GV). **(B, C)** Spatial localization of extremal gradient values (top/last 10%) for SG1 (B) and SG2 (C), overlaid on the surface in the left cerebral cortex. **(D)** Correlation spectra quantify absolute Pearson coefficients (| *r* |) between the first 100 geometric eigenmodes and SG1/SG2 in the tree shrew. Blue circles identify peak GGC values, defined as the maximal coupling strength between each gradient and its optimally correlated geometric eigenmode. **(E)** Surface mappings of the peak GGC relationships identified in (D) for SG1 (aligned with Mode 3) and SG2 (aligned with Mode 2) in the tree shrew cerebral cortex. **(F)** Scatterplots demonstrate a robust positive correlation (*r* = 0.847) between the peak GGC strength (| *r* | values from D) and gradient-specific explained variance. **(G)** Cross-domain quantification of peak GGC strength. Absolute Pearson correlations (| *r* |) represent peak GGC (maximal coupling strength between region-specific gradients (structural/functional) and their optimally correlated geometric eigenmodes). Values annotate the maximal | *r* | per gradient-domain combination with corresponding eigenmode indices (e.g., SG1-Mode3 for left cortex of the tree shrew: | *r* | = 0.78). **(H)** Domain-wise GGC-variance correlations. Each point represents the Pearson correlation coefficient (*r*) quantifying the association between peak GGC strength and gradient-explained variance within a specific species-brain region-imaging modality combination. The red dashed line marks the grand mean (*r* = 0.797) across 16 experimental units, demonstrating consistent geometry-driven constraints on neurobiologically critical gradients. (L, left hemisphere; R, right hemisphere). Results for right cerebral cortex of the tree shrew are presented in S8 Fig. The data underlying this Figure can be found in S1 Data.

Initial analysis of structural connectivity gradients in the tree shrew left cerebral cortex revealed distinct spatial patterns that appeared visually coupled to cortical folding geometry (Fig 6A–6C). Specifically, SG1 (13.7% of variance) spanned from the medial parietal cortex to the prefrontal and lateral temporal cortices, while subsequent gradients (SG2–SG4) exhibited distinct topographic progressions. Quantitative validation via Principal Gradient analysis confirmed that the top and bottom 10% of SG1–SG2 loading voxels were preferentially localized to geometric curvature vertices (Fig 6B and 6C), motivating a rigorous spectral comparison.

We quantified the spatial similarity between the first 100 long-wavelength geometric eigenmodes (Mode 1–100) and SGs, revealing significant GGC in the primary gradients (SG1–SG2) across the left cerebral cortex (|*r*| ≥ 0.75) (Fig 6D). Consistent with observations in human subcortical structures [32], low-frequency eigenmodes effectively captured the dominant connectivity gradients: SG1 showed strong coupling with Mode 3 (|*r*| = 0.78), while SG2 aligned closely with Mode 2 (Fig 6E). The specificity of these couplings was confirmed by their significantly higher correlation strengths compared to alternative pairings (Fig 6D), indicating that eigenmodes selectively encode connectome gradients rather than reflecting random associations. Furthermore, we observed a robust positive correlation between GGC strength and gradient variance (*r* = 0.847; Fig 6F), demonstrating that functionally critical gradients (i.e., those explaining higher variance) are subject to stronger geometric constraints. The biological validity of these findings is reinforced by a remarkable inter-hemispheric symmetry (S8 Fig). Contralateral analyses confirmed that primary and secondary gradients of both hemispheres independently aligned with identical geometric modes (e.g., SG1 to Mode 3), yielding comparable GGC strengths (|*r*| ≥ 0.63). This precise correspondence was verified by Spin Tests (10,000 permutations) to significantly exceed null distributions (S9 Fig), confirming that the observed coupling reflects a genuine topological constraint rather than a spurious association driven by spatial autocorrelation.

To assess generalizability, we extended these analyses to cerebellar and hippocampal regions, integrating data from humans, macaques, marmosets, and mice [20,23,25,26,64], as well as fMRI-derived FGs. Our results revealed a strikingly conserved GGC across all tested species, modalities, and brain regions (Figs 6G and S10): both structural and FGs exhibited robust alignment with geometric eigenmodes (all |*r*| ≥ 0.38), with the first 8 eigenmodes capturing the dominant organizational patterns. Crucially, these couplings were statistically validated against spatial autocorrelation-preserving null models (Spin Tests, 10,000 permutations; S9 Fig).

Finally, a consistent positive relationship between GGC strength and gradient variance (*r* ≥ 0.70) was observed across nearly all modalities, with the tree shrew hippocampus representing the sole moderate exception (left: *r* = 0.46; right: *r* = 0.64) (Fig 6H). This robust association is particularly striking given the distinct methodological origins of the metrics involved: connectivity gradients are derived from complex diffusion/functional MRI pipelines, whereas geometric eigenmodes are mathematical constructs derived solely from surface topology [32]. This convergence underscores the universal role of brain geometry in constraining FGs across phylogenetic scales—from mice to primates—highlighting a consistent prioritization of alignment with functionally critical, high-variance axes.

## Discussion

In this study, we established a comprehensive atlas of the tree shrew brain using ultra-high-resolution MRI, delineating fine-scale neuroanatomical and connectivity profiles that bridge the rodent-primate gap.

Unlike previous studies restricted to basic regional analyses [16–19], our high-resolution approach resolved complex cerebellar foliation and hippocampal architectures, revealing that the tree shrew is not merely an intermediate form. Instead, our findings demonstrate that the tree shrew represents a distinct evolutionary mosaic, characterized by the decoupling of cerebellar and hippocampal adaptations. Specifically, the cerebellum exhibits a striking juxtaposition of ecological and phylogenetic signatures. Its massive volumetric expansion parallels that of the red-bellied tree squirrel (*Callosciurus* *erythraeus*), another agile arboreal climber, which we found to exhibit a similarly high relative cerebellar volume (~16.3%, S11 Fig). This convergent expansion suggests that the cerebellar enlargement in the tree shrew is primarily an ecological adaptation to the complex sensorimotor demands of three-dimensional navigation, rather than a strictly primate-like trait. However, internally, the cerebellar functional architecture mirrors that of primates: the emergence of the SG4 gradient (recapitulating the primate motor-to-non-motor axis) indicates that the tree shrew has evolved sophisticated connectional circuitries that align with the primate lineage.

In contrast, the hippocampus retains a rodent-like architectural scaling, preserving ancestral subfield proportions, yet its FGs encode an evolutionarily conserved longitudinal axis that aligns across species. Meanwhile, the cerebral cortex presents a transitional phenotype, exhibiting morphological metrics (such as volume and gyrification) that scale linearly between rodents and primates. This mosaic organization implies that brain evolution proceeds through modular adaptations, where distinct neural systems evolve at different rates and under different pressures (ecological versus phylogenetic) to meet specific survival demands.

Beyond these specific regional adaptations, our multidimensional analysis reveals a striking dichotomy between morphological mosaicism and organizational conservation. On one hand, the neuroanatomical landscape is defined by a unique evolutionary mosaic. Unlike a simple linear transition from rodent to primate, the tree shrew brain integrates distinct adaptive strategies: the cerebellum has expanded convergently with arboreal squirrels to support climbing (an ecological adaptation), whereas the hippocampus retains a conserved rodent-like architecture (a phylogenetic retention). This confirms that brain evolution in the tree shrew is modular, driven by specific survival demands rather than a uniform global scaling. In stark contrast, the GGC emerged as an evolutionarily invariant principle. Despite these diverse morphological origins—whether a structure expanded for locomotion or remained ancestral—the tight spatial alignment between geometric eigenmodes and connectivity gradients remained robust. This dissociation suggests that while evolutionary pressures drove the physical remodeling of specific neural substrates (creating the mosaic anatomy), the fundamental biophysical constraints governing functional organization (GGC) have remained fixed. The strong correlation between coupling strength and gradient variance further suggests that neurobiologically significant gradients are hierarchically prioritized to anchor to brain geometry, serving as a conserved “morphological code” throughout mammalian evolution.

The conserved GGC likely reflects an underlying molecular scaffold. Given the alignment between gene expression and connectivity gradients observed in other species [26,28,65,66], we hypothesize that the tree shrew harbors primate-like transcriptomic profiles in the cerebellum (aligning with its expanded gradients) but rodent-like signatures in the hippocampus. Resolving the interplay between genetic patterning, geometric constraints, and connectivity gradients remains a fundamental challenge [32,66–69]. Our findings suggest these factors form an interdependent system: genes sculpt the geometry, which in turn constrains the connectivity hierarchy. The transitional phylogenetic position of the tree shrew provides a unique window to decouple these mechanisms, offering a roadmap to unravel how evolution balances functional innovation (e.g., cerebellar rewiring) with structural robustness (e.g., conserved GGC).

Several limitations warrant consideration. First, the sample size (*n* = 11) limits the generalizability of population-averaged templates. Second, despite ultra-high resolution, diffusion tensor modeling has intrinsic limitations in resolving crossing fibers [70–72]. Although diffusion tensor imaging demonstrates spatial concordance with neural tracing techniques [73,74], rigorous validation employing viral-based anterograde/retrograde tracing [75,76], remains essential to authenticate identified neuroanatomical gradients. Third, while structural and functional connectivity have shown significant correspondence [77–79], reliance on SGs as proxies for functional hierarchies remains unconfirmed. Future acquisition of tree shrew-specific awake fMRI data is required to directly assess structure-function coupling, especially in the cerebellar and hippocampal regions where ecological adaptations likely drive specialized functions.

Collectively, this study establishes the tree shrew as an evolutionary mosaic, blending conserved ancestral traits with derived neuroanatomical adaptations. This resource bridges a critical gap in comparative neuroanatomy, providing an indispensable framework for deciphering divergence at the rodent-primate transition. By integrating this resource with spatial transcriptomics and biomechanical modeling [80–82], future research can resolve a central evolutionary question: does neuroanatomical diversity arise from incremental adjustments to a conserved genetic-geometric scaffold, or through the active rewiring of connectivity within existing bounds?

## Materials and methods

### Experimental design

The objective of this study was to construct a high-resolution, MRI atlas of the tree shrew brain to delineate its neuroanatomical architecture and characterize whole-brain structural connectivity gradients, thereby establishing a foundational resource for cross-species evolutionary comparisons. The study was designed to integrate ultra-high-field structural and diffusion-weighted imaging data from ex vivo tree shrew brains. Prespecified components included: 1) the acquisition of T2*-weighted anatomical and multi-shell diffusion MRI datasets; 2) the construction of a population-averaged neuroanatomical template; 3) the development of a hybrid manual-automated parcellation protocol to define macroscale brain regions, cerebellar lobules, and hippocampal subfields; 4) the reconstruction of cortical, cerebellar, and hippocampal surfaces for geometric eigenmode analysis; 5) the mapping of structural connectivity gradients using diffusion tractography and nonlinear dimensionality reduction; and 6) a predefined comparative analysis of cerebellar organization and connectivity gradients across multiple species (mouse, marmoset, macaque, human, and tree shrew) using publicly available datasets. All imaging, processing, and analytical workflows were predetermined prior to data collection to ensure consistency and reproducibility.

### Animal preparation and MRI data acquisition

Eleven adult tree shrews (*Tupaia belangeri chinensis*; 9 males, 2 females; mean age = 2 years) were intramuscularly anesthetized with ketamine (50 mg/kg) and pentobarbital (60 mg/kg). Transcardial perfusion was sequentially performed using ice-cold phosphate-buffered saline (PBS, pH 7.4) followed by 4% paraformaldehyde (PFA) in 0.1 M phosphate buffer. Intact brains with skulls were post-fixed in 4% PFA containing 0.3% (w/v) gadolinium-based contrast agent (Gd-DTPA, Magnevist, Bayer) for 21 days at 4 °C, then transferred to PBS solution supplemented with 0.3% Gd-DTPA for additional 7 days to enhance magnetic resonance contrast. All experimental procedures were approved by the Animal Ethics Committee of the Kunming Institute of Zoology, Chinese Academy of Sciences (Protocol ID: IACUC18027).

Anatomical and diffusion-weighted datasets were acquired on a Bruker BioSpec 9.4T horizontal bore scanner (ParaVision 6.0.1) equipped with a quadrature volume transmit/receive coil. For whole-brain structural imaging, a T2*-weighted sequence was optimized with repetition time (TR) = 20 ms, echo time (TE) = 5.8 ms, and flip angle = 15°, achieving isotropic voxel dimensions of 50 × 50 × 75 μm through a 22 × 33 × 18 mm field of view (440 × 660 × 240 matrix). The sequence incorporated Hamming-filtered k-space sampling with zero-fill interpolation (Z-factor = 1.34), yielding 11 complete brain acquisitions at 29.6 min per scan.

Diffusion tensor imaging employed a spin-echo echo-planar sequence (TR/TE = 200/28.96 ms) with three-shell encoding: *b* = 50 s/mm^2^ (6 directions), 1,500 s/mm^2^ (30 directions), and 3,000 s/mm^2^ (60 directions). A 16-segment EPI readout with 250 kHz bandwidth and 125 µm isotropic resolution ensured whole-brain coverage (22 × 33 × 18 mm FOV) while mitigating eddy current distortions. Monopolar diffusion gradients (δ = 3.2 ms, Δ = 12 ms) were applied along vectors optimized through electrostatic repulsion modeling. The 12-hour 8-min acquisition included eight dummy scans for signal stabilization and balanced spoiler gradients (50 mT/m) for residual magnetization suppression.

### Population-averaged template and parcellation atlas

A population-averaged neuroanatomical template was constructed through iterative multi-scale registration with the best-scanned individual as the base using “antsMultivariateTemplateConstruction2.sh” command of Advanced Normalization Tools (ANTs) [83]. Diffusion tensor data from a single specimen were registered to template space by affine and non-linear registration, using the “antsRegistrationSyN.sh” command of ANTs. A hybrid manual segmentation protocol, guided by a template-based approach, delineated 20 macroscale brain regions alongside 16 cerebellar lobules and 5 hippocampal subregions according to the definitive brain atlas [34]. Three independent raters validated cytoarchitectonic boundaries through consensus-based delineation of primary cerebellar fissures, while hippocampal subfield segmentation adhered to stratum pyramidale cytoarchitectural discontinuities. The brain anatomical regions are including: cerebral cortex (Cortex), cerebral white matter (Wm), amygdala (Amy), thalamus (Thal), medial geniculate nucleus (MGN), lateral geniculate nucleus (LGN), caudate (Cd), putamen (Pu), nucleus accumbens (Acb), claustrum/endopiriform claustrum (Cl), hypothalamus (Hypo), septum (Sep), globus pallidus (GP), inferior colliculus (IC), superior colliculus (SC), periaqueductal gray (PAG), substantia nigra (SNR), hippocampus (Hip), cerebellum (Ceb), and olfactory bulb (Olf). Cerebellar parcellation followed anatomical landmarks: lingula I (I), central lobule II (II), culmen III (III), declive IV (IV), lobule V (V), folium VI (VI), tuber VII (VII), pyramid VIII (VIII), uvula IX (IX), nodulus X (X), simplex lobule (SIM), paramedian lobule (Par), copula (Cop), ﬂocculus (Fl), paraﬂocculus (PFI), Crus I, and Crus II. Hippocampal subregions included cornu ammonis1 (CA1), cornu ammonis2 (CA2), cornu ammonis3 (CA3), dentate gyrus (DG), and subiculum (Sub). To explore potential sex differences despite the limited and unbalanced female sample (*n* = 2 females, *n* = 9 males), we conducted a supplemental analysis using the 95% confidence interval (CI) of the male group as a normative reference. For each brain region, the mean and 95% CI were calculated from the male participants’ data. A female participant’s value was considered to deviate from the male normative range if it fell outside this interval. This approach enabled identification of brain regions where individual female brains exhibited patterns distinct from the male group, providing preliminary insights into sex-specific variations despite the small female sample.

### Surface reconstruction and geometric eigenmode analysis

Cortical, cerebellar, and hippocampal surfaces were reconstructed using our optimized multimodal pipeline integrating AFNI (v24.3.06) [84], FSL (v6.0.4) [85], and FreeSurfer (v3.0) [42]. For cerebellar surface modeling, white matter (WM) and gray matter (GM) segmentations were derived through semi-automated protocols combining template-guided initialization and manual cytoarchitectonic refinement. T2*-weighted images and tissue probability maps were resampled to a standardized right-superior-posterior (RSP) orientation. Image headers were modified to 100 μm isotropic resolution (original voxel size: 50 × 50 × 75 μm^3^; no spatial downsampling applied) to align with stereotaxic coordinates. GM and WM masks were intensity-normalized (80 and 110, respectively) and smoothed (gaussian kernel: 0.6 mm FWHM) to synthesize T1-weighted contrast. Initial WM surfaces were tessellated using FreeSurfer’s topology-preserving algorithms (“mri_tessellate”, “mris_extract_main_component”). Pial surfaces were iteratively refined via a modified “mris_make_surfaces” workflow, incorporating curvature regularization (*λ* = 0.0005) to resolve GM–WM boundary ambiguities inherent to cerebellar foliation. Surface meshes were smoothed (“mris_smooth”) and manually corrected to eliminate topological inaccuracies arising from foliar complexity. Cortical surfaces were reconstructed using analogous workflows but tailored parameters: Gaussian smoothing (FWHM = 6 mm), and curvature regularization (*λ* = 0.1). Hippocampal surfaces, minimal smoothing (FWHM = 0.1 mm), and directly utilized the initial mesh (xx.orig) as the pial boundary without iterative refinement. All surfaces were visually inspected and computationally validated against raw histoarchitectonic landmarks to ensure geometric fidelity. Topologically corrected meshes were generated for cerebral hemispheres (left: 88,078 vertices; right: 90,462 vertices), hippocampi (left: 28,408; right: 27,442 vertices), and cerebellum (129,828 vertices). Volumetric cerebellar lobule and hippocampal parcellations were mapped to surfaces using “wb_command -volume-to-surface-mapping” method of Connectome Workbench [86]. The “wb_command -surface-vertex-areas” tool was used to calculate the regional surface areas.

We quantified surface geometry across cortical, hippocampal, and cerebellar regions by computing eigenmodes—mathematical descriptors of intrinsic shape patterns, using the Laplace-Beltrami operator on triangular surface meshes. The LaPy Python library [87,88] solved the Helmholtz equation for each structure’s mesh, generating eigenmodes that hierarchically encode curvature variations, from fine-scale folds (e.g., cerebellar folia) to global curvature gradients. Cubic finite-element discretization ensured numerical stability for highly folded surfaces. Eigenmodes were iteratively refined until spectral convergence, providing a multiscale representation of neuroanatomical shape.

### Structural connectivity gradient mapping

Diffusion-weighted images were preprocessed using “dwidenoise” of MRtrix3 [89] for Rician noise reduction and FSL’s spline-interpolated eddy current correction. Our dataset included two shells: *b* = 1,500 s/mm^2^ (30 directions) and *b* = 3,000 s/mm^2^ (60 directions). For fiber orientation distribution (FOD) estimation, we focused the Tournier algorithm on the high-angular-resolution *b* = 3,000 s/mm^2^ shell, which provides optimal angular sampling density and signal-to-noise ratio for resolving complex crossing fibers—a critical requirement for the dense neural architecture of the tree shrew. Tensor-derived metrics (FA, eigenvectors) were computed via FSL’s “dtifit” with *b*-values/*b*-vectors averaged across acquisitions. As part of quality control, we visually inspected the V1-V3 components (where V1 represents the principal diffusion direction and V2/V3 the orthogonal transverse directions) to ensure directional coherence of fiber orientation estimates, establishing a high-fidelity anatomical foundation prior to tractography (S1A Fig). FODs were estimated using constrained spherical deconvolution (MRtrix3 dwi2response/dwi2fod; response function: tournier algorithm) and visually verified for directional coherence of V1–V3 components.

Whole-brain probabilistic tractography (streamlines: 1,000,000 tracts, step size: 0.2 mm, angular threshold: 45°, FOD amplitude cutoff: 0.1) was performed with “tckgen” command of MRtrix3. To construct the structural connectome, we first defined anatomical boundaries (cortex, cerebellum, hippocampus) based on stereotaxic coordinates and anatomical delineations from Zhou and Ni [34]. These regions were further subdivided using K-means clustering on 3D spatial coordinates, generating spatially coherent and isotropic micro-clusters as nodes (S12 Fig). This approach ensures that the connectivity matrix captures local anatomical proximity and continuous gradient transitions without confounding from global geometric variance of large regions. Structural connectomes were generated via “tck2connectome” using the parcellation label as nodes, with streamline assignments mapped to a node-parcellated adjacency matrix. Affinity matrices were derived by computing the Pearson correlation between the connectivity profiles of all pairs of nodes. To ensure cross-species comparability, we then applied the Normalized Angle kernel—a scale-invariant measure that focuses on topological similarity rather than absolute connection magnitudes, which is essential for comparing species with vastly different brain sizes. Diffusion map embedding was subsequently performed via BrainSpace (v0.1.2) [64] to identify structural connectivity gradients. This analytical framework has been widely validated across mammals [20,26], enabling direct placement of tree shrew gradients within the broader mammalian evolutionary context.

All gradients were computed in each subject’s native space to preserve anatomical precision, then registered to the template space via “antsRegistrationSyN.sh” (ANTs) for visualization and cross-species comparison. This “calculate-natively, register-laterally” approach minimizes spatial distortions and smoothing artifacts. The gradient results were registered to the template space via “antsRegistrationSyN.sh” command of ANTs and mapped to cortical/hippocampal/cerebellar surfaces using Connectome Workbench’s “wb_command -volume-to-surface-mapping”. Surface-based gradients underwent an isotropic smoothing (FWHM: cerebellum = 0.2 mm, hippocampus = 0.2 mm, cortex = 0.3 mm) to suppress noise while preserving laminar/foliar geometry.

To validate the robustness of this approach, we performed a secondary sensitivity analysis using the Dhollander-based multi-shell multi-tissue CSD (MSMT-CSD) method, which incorporates all shells and tissue compartments (WM, GM, CSF). For the tree shrew cerebral cortex, the resulting SGs were highly convergent with our original findings across the first two gradients (*r* = 0.87–0.90, *p* < 0.0001), confirming that the *b* = 3,000 shell captures essential directional information and that our conclusions are robust across estimation algorithms (S13 Fig).

### Cross-species comparative analysis

The data for cross-species comparative analysis were obtained from published resources. The human neuroimaging templates and cortical functional connectivity gradients from Human Connectome Project (HCP) [90]. The diffusion MRI data of human were acquired from the OpenNeuro dataset (https://doi.org/10.18112/openneuro.ds004910.v1.0.1). Macaque templates and diffusion MRI data were obtained from the NIMH Macaque Template (NMT) repository [43] and a high-resolution ex vivo macaque resource, respectively. Marmoset anatomical templates and cortical functional connectivity gradients were obtained from the Marmoset Brain Mapping Project [12,41] and a prior marmoset study [23]. The mouse data were obtained from a group-averaged atlas [44]. The cerebellar functional connectivity gradients for the mouse, marmoset, and macaque were derived from our prior comparative studies [25,26]. For cross-species comparisons of cerebellar subregional volumes, lobules I and II were merged into I-II, and lobules IV and V into IV-V, establishing 15 standardized subregions across species. Pearson correlation coefficients were computed to quantify interspecies similarity in the proportional volumes of these cerebellar subregions. When assessing regional-averaged GVs, cerebellar hemispheres were differentiated, resulting in 30 distinct partitions (15 subregions × 2 hemispheres). Interspecies similarity of cerebellar gradients was then evaluated using Pearson correlations across these partitions.

For GGC analyses, all available gradients were incorporated except for marmoset cortical FGs, where only the first 20 gradients were accessible. These top 20 components capture the vast majority of cumulative variance (87.9%) and reflect macroscale organizational principles, while higher-order gradients predominantly represent local noise. To assess whether the selected number of gradients (*k*) biased our results, we performed sensitivity analyses on marmoset cortex using both functional and SGs (S14 Fig). For functional data, we varied *k* from 10 to 20 (the stable range for FGs); for structural data, we extended *k* up to 69 components. Correlation coefficients remained highly stable across all tested ranges for both modalities, demonstrating that GGC metrics are driven by fundamental organizational axes and are insensitive to the specific gradient cutoff threshold. This confirms that the limitation of accessible gradients in fMRI does not affect the validity of our comparative conclusions.

### Statistical validation of geometry–gradient coupling

To rigorously validate the spatial correspondence between geometric eigenmodes and connectivity gradients, we employed a Spin Test (spatial permutation) approach that preserves the intrinsic spatial autocorrelation of the data [91]. Standard correlation methods are prone to Type I errors due to the smooth, continuous nature of neuroimaging data; therefore, we generated null models by randomly rotating the spherical representation of the cortical/cerebellar surfaces.

For each hemisphere and species, we performed the following steps: (1) Spherical Projection and Rotation: The original surface vertices were mapped to a sphere. We generated 10,000 random rotation matrices uniformly sampled from the group of 3D rotations. (2) Data Permutation: These rotation matrices were applied to the spherical coordinates of the original surface. For each permutation, the original connectivity GVs were remapped to the nearest neighbor on the rotated sphere. This process effectively scrambled the anatomical location of the GVs while preserving their spatial relationships (autocorrelation) and distribution. (3) Null Distribution Generation: For every geometric eigenmode, we calculated the Spearman rank correlation between the eigenmode and the 10,000 permuted gradient maps. This created a specific null distribution of correlation coefficients for each mode-gradient pair. (4) Significance Testing: The empirical (observed) correlation was compared against this null distribution. The permutation p-value (*P*_spin_) was calculated as the proportion of null correlations with an absolute magnitude greater than or equal to the observed absolute correlation:


$$P_{\text{spin}}=\frac{N_{\left|r_{\text{null}}\right|\geq \left|r_{\text{obs}}\right|}+\text{1}}{N_{\text{perm}}+\text{1}}$$

where *N*_perm_ = 10,000.

## Supporting information

S1 Fig Multi-scale neuroanatomical templates and regional subdivisions. **(A)** Principal Diffusion Direction (V1) map. This panel illustrates the primary eigenvector (V1), representing the dominant water diffusion direction as a proxy for axonal orientation. The magnified inset highlights the corpus callosum, where the predominant left-right orientation is visualized in red, consistent with established commissural fiber trajectories. **(B)** Overview of the tree shrew brain atlas online interface (http://www.treeshrewdb.org/MRI/). **(C)** Cerebellar subdivisions. Parcellation of the cerebellum based on consensus cytoarchitectonic boundaries at cerebellar fissures. The white dashed box indicates the area magnified in the inset, with red lines denoting the specific fissures that separate cerebellar lobules. **(D)** Hippocampal subdivisions. Segmentation of the hippocampus based on cytoarchitectural discontinuities. The white dashed box indicates the region magnified in the inset. Red arrows point to distinct borders between hippocampal subfields, with color-coded boundaries representing the final subregional partitions. **(E–G)** Volumetric distributions. Template-derived volume distributions across different anatomical hierarchies: **(E)** whole-brain partitions, **(F)** cerebellar lobules, and **(G)** hippocampal subregions. The abbreviations are defined in the Materials and Methods section. The data underlying this Figure can be found in S1 Data.
(TIF)

S2 Fig Volumetric and morphometric characteristics of the tree shrew brain parcellation. **(A)** Consistency between template and individual-level parcellations. Comparison of spatial alignment and boundary definitions between the population-averaged template and individual-level brain partitions. **(B)** Individual-level volumetric distributions of brain regions. Quantitative distribution of brain region volumes across the cohort. Insets highlight the cerebellar lobules (*upper right*) and hippocampal subdivisions (*lower left*). Red dots denote population means across all individuals. **(C–E)** Inter-subject volumetric variability. Coefficient of variation (CV) illustrating the degree of inter-subject variability in individual-level volumes across different anatomical scales: **(C)** whole-brain partitions, **(D)** cerebellar lobules, and **(E)** hippocampal subregions. The data underlying this Figure can be found in S1 Data.
(TIF)

S3 Fig Sex differences in absolute and relative volumes of brain regions. **(A)** Absolute volume. **(B)** Relative volume (normalized to total brain volume). The y-axis represents the ratio relative to the male mean for each brain region. Gray horizontal lines (or black error bars) indicate the 95% confidence intervals (CI) for males, while red dots represent the values for females. The data underlying this Figure can be found in S1 Data.
(TIF)

S4 Fig Comparative analysis of brain and body metrics across species. **(A)** Comparison of relative brain region volumes across species. **(B)** Absolute brain volume-to-body mass ratios across species, calculated using representative body weights for each species (mouse, 35 g; tree shrew, 130 g; marmoset, 400 g; macaque, 8,000 g). The data underlying this Figure can be found in S1 Data.
(TIF)

S5 Fig Structural connectivity gradients of the tree shrew cerebellum. **(A–D)** Gradient values for gradients 1–4, displayed as a box plot (median and interquartile range, IQR) across cerebellar lobules. The data underlying this Figure can be found in S1 Data.
(TIF)

S6 Fig Structural and functional connectivity gradients alignment in cerebellum. **(A–F)** The gradients similarity between structural and functional connectivity gradients (SG1–2 and FG1–2) in the macaque **(A, B)**, marmoset **(C, D)**, and mouse **(E, F)**. The similarity was quantified by Pearson correlation coefficients (*r*) based on the regional-averaged-GV with hemispheric differentiation. The data underlying this Figure can be found in S1 Data.
(TIF)

S7 Fig Structural connectivity gradients of tree shrew right hippocampus. **(A–D)** Mirroring Fig 5B–E for the right hippocampus. **(A)** Spatial alignment of SG1 and SG2 values along the longitudinal axis (DV-axis). **(B)** DV-axis progression of SG1 and SG2 values, color-mapped by DV-axis position. **(C)** Subregional heterogeneity of SG1 and SG2 across hippocampal subregions (color-coded). **(D)** Spatial localization of extremal gradient values (top/last 10%) for SG1 and SG2. The data underlying this Figure can be found in S1 Data.
(TIF)

S8 Fig Structural connectivity gradients of the tree shrew right cerebral cortex. **(A–F)** Mirroring Fig 6A-F for the right cerebral cortex. **(A)** Cortical surface maps of the first four structural gradients (SG1–SG4) in the right hemisphere, with color-encoded by gradient value (GV). **(B, C)** Spatial localization of extremal gradient values (top/last 10%) for SG1 **(B)** and SG2 **(C)**, overlaid on the surface in the right hemisphere. **(D)** Correlation spectra quantify absolute Pearson coefficients (| *r* |) between the first 100 geometric eigenmodes and SG1/SG2 in the tree shrew. Blue circles identify peak GGC values, defined as the maximal coupling strength between each gradient and its optimally correlated geometric eigenmode. **(E)** Surface mappings of the peak GGC relationships identified in (D) for SG1 (aligned with Mode 3) and SG2 (aligned with Mode 2) in the tree shrew cerebral cortex. **(F)** Scatterplots demonstrate a robust positive correlation (*r* = 0.638) between the peak GGC strength (| *r* | values from **D**) and gradient-specific explained variance. The data underlying this Figure can be found in S1 Data.
(TIF)

S9 Fig Inter-hemispheric and cross-species consistency of geometry–gradient coupling (GGC). **(A, B)** Spatial profiles of the first two structural gradients (SG1, SG2) in the left and right cortical hemispheres of the tree shrew **(A)** and marmoset **(B)**. **(C)** Structural (SG1–2) and functional (FG1–2) gradients in the mouse cerebellum. Statistical significance of spatial correspondence was assessed using a Spin Test (spatial permutation) preserving spatial autocorrelation. For each hemisphere and species, 10,000 random spherical rotations were applied to remap gradient values while preserving their spatial structure. For each geometric eigenmode, Spearman rank correlation was computed against the 10,000 permuted gradient maps to generate a null distribution. The permutation *p*-value (*P*_*spin*_) was calculated as the proportion of null correlations with absolute magnitude ≥ the observed absolute correlation. The data underlying this Figure can be found in S1 Data.
(TIF)

S10 Fig Conserved peak geometry–gradient coupling (GGC) across phylogenetically diverse species, neuroanatomical systems, and imaging modalities. **(A–N)** Surface mappings of representative peak GGC relationships across domains: **(A, B)** Structural gradients for the left **(A)** and right **(B)** cerebral cortex in the marmoset. **(C, D)** Structural gradients for the left **(C)** and right **(D)** cerebral cortex in human. **(E, F)** Cortical functional gradients for the left cerebral cortex in the marmoset **(E)** and human **(F)**. **(G–J)** Cerebellar structural gradient in mouse **(G)**, tree shrew **(H)**, marmoset **(I)**, and macaque **(J)**. **(K)** Hippocampal structural gradient (only the GGC values of the left hippocampus was shown). **(L–N)** Cerebellar functional gradients in the mouse **(L)**, marmoset **(M)**, and macaque **(N)**. Absolute Pearson correlations (| *r* |) represent peak GGC (maximal coupling strength between region-specific gradients (structural/functional) and their optimally correlated geometric eigenmodes). The data underlying this Figure can be found in S1 Data.
(TIF)

S11 Fig Comparative visualization of cerebellar expansion in tree shrew and red-bellied tree squirrel. **(A)** Tree shrew. **(B)** Red-bellied tree squirrel. The cerebellar regions are highlighted in red, demonstrating the prominent cerebellar volume relative to the whole brain in both arboreal species. The relative cerebellar volume is approximately 13.4% in the tree shrew and 16.3% in the red-bellied tree squirrel. The data for the red-bellied tree squirrel are openly available as part of the tree shrew database at http://www.treeshrewdb.org/MRI/.
(TIF)

S12 Fig Anatomical parcellation via spatially-constrained K-means clustering. The brain was parcellated into *N* = 400 distinct regions based on spatial coordinates. Panels illustrate the parcellation results for the **(A)** cortex, **(B)** cerebellum, and **(C)** hippocampus, respectively. This coordinate-based clustering ensures the spatial contiguity and structural integrity of each resulting parcel.
(TIF)

S13 Fig Cross-validation of structural gradient estimation using Tournier and Dhollander algorithms in the tree shrew cerebral cortex. The primary gradients are shown for the left **(A)** and right hemispheres **(B)**. Spatial similarity between the gradient maps derived from the two algorithms was quantified using Pearson correlation coefficients, where *r* represents the correlation strength between the two gradient maps and *p* indicates the statistical significance of this correlation. The high spatial correspondence (left hemisphere: *r* = 0.90, *p* < 0.0001; right hemisphere: *r* = 0.87, *p* < 0.0001) demonstrates robust estimation across algorithms and consistent bilateral organizational patterns. The data underlying this Figure can be found in S1 Data.
(TIF)

S14 Fig Sensitivity analysis of GGC stability across gradient numbers (*k*). Quantitative validation of the robustness of Geometry-Gradient Correlation (GGC) across varying numbers of gradient components. **(A)** Functional gradients: The correlation between peak GGC strength and explained variance remains highly stable within the effective range of functional components (*k* = 10–20). **(B)** Structural gradients: To test the limits of stability, diffusion MRI data were analyzed across a broader spectrum (*k* = 10–69). The GGC metric shows remarkable robustness even as higher-order components are included, with no significant degradation or drift in correlation coefficients. The data underlying this Figure can be found in S1 Data.
(TIF)

S1 Table The volume of 20 brain regions. (DOCX)

S2 Table The volume of 16 cerebellar lobules. (DOCX)

S3 Table The volume of 5 hippocampal subregions. (DOCX)

S4 Table The surface area of 16 cerebellar lobules. (DOCX)

S5 Table Summary of regional brain metrics across species. (DOCX)

S1 Data Underlying numerical data for all main and supplementary figures. (XLSX)

## Abbreviations

ANTs: Advanced Normalization Tools
AP: anteroposterior
CI: confidence interval
DG: dentate gyrus
FG: functional gradient
FOD: fiber orientation distribution
GGC: geometry–gradient coupling
GI: gyrification index
GM: gray matter
GV: gradient value
MRI: magnetic resonance imaging
PBS: phosphate-buffered saline
PFA: paraformaldehyde
PFL: paraflocculus
SGs: structural gradients
TE: echo time
TR: repetition time
WM: white matter
