# Supplementary figures and images for: An anatomical and connectivity atlas of the tree shrew brain to bridge rodent and primate neuroanatomy
Source: PLoS Biol. 2026 May 4;24(5):e3003773. doi: 10.1371/journal.pbio.3003773 (PMC13138645; doi:10.1371/journal.pbio.3003773)

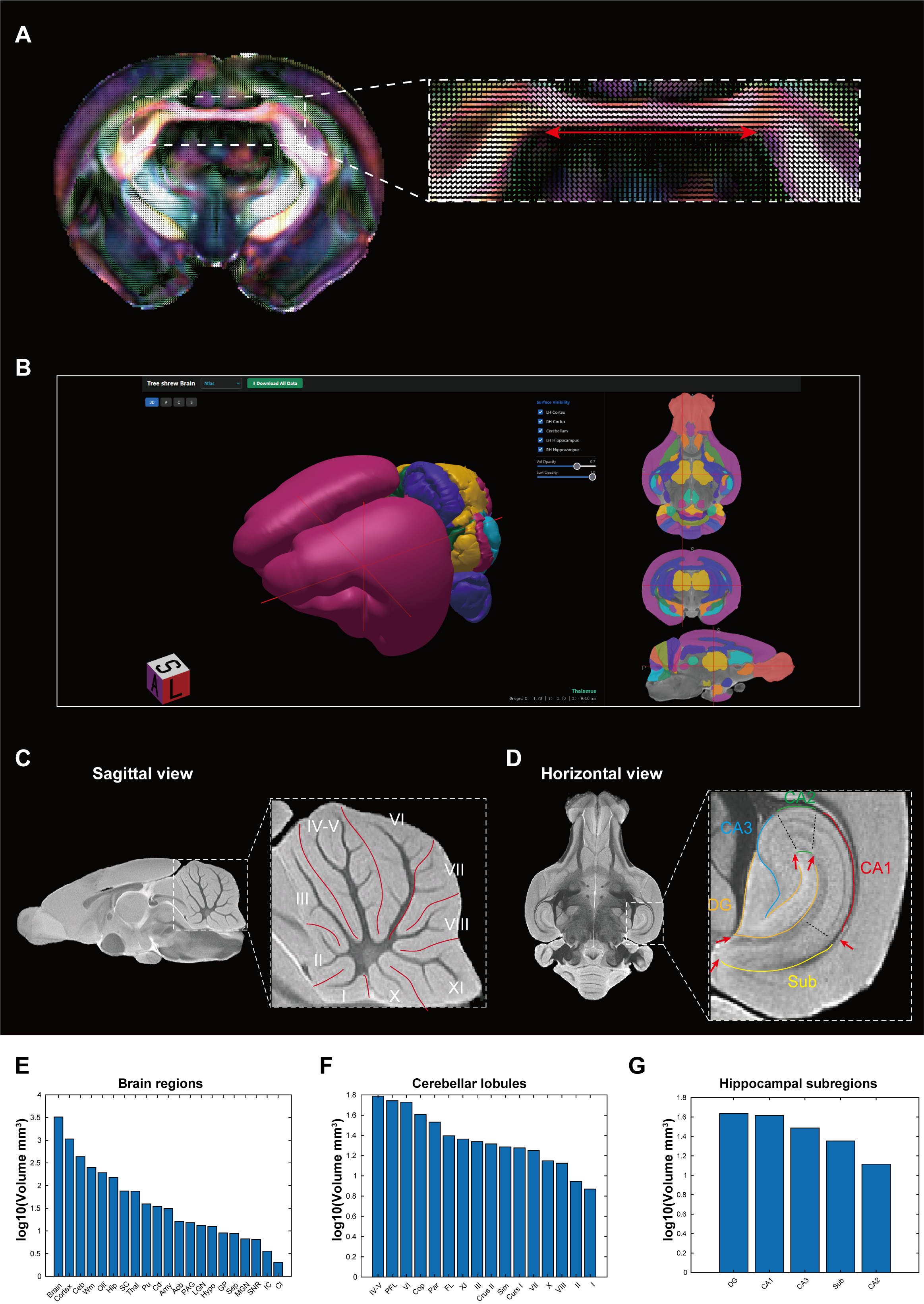

Supplement: S1 Fig — (A) Principal Diffusion Direction (V1) map. This panel illustrates the primary eigenvector (V1), representing the dominant water diffusion direction as a proxy for axonal orientation. The magnified inset highlights the corpus callosum, where the predominant left-right orientation is visualized in red, consistent with established commissural fiber trajectories. (B) Overview of the tree shrew brain atlas online interface (http://www.treeshrewdb.org/MRI/). (C) Cerebellar subdivisions. Parcellation of the cerebellum based on consensus cytoarchitectonic boundaries at cerebellar fissures. The white dashed box indicates the area magnified in the inset, with red lines denoting the specific fissures that separate cerebellar lobules. (D) Hippocampal subdivisions. Segmentation of the hippocampus based on cytoarchitectural discontinuities. The white dashed box indicates the region magnified in the inset. Red arrows point to distinct borders between hippocampal subfields, with color-coded boundaries representing the final subregional partitions. (E–G) Volumetric distributions. Template-derived volume distributions across different anatomical hierarchies: (E) whole-brain partitions, (F) cerebellar lobules, and (G) hippocampal subregions. The abbreviations are defined in the Materials and Methods section. The data underlying this Figure can be found in S1 Data. (TIF) [file pbio.3003773.s001.tif]

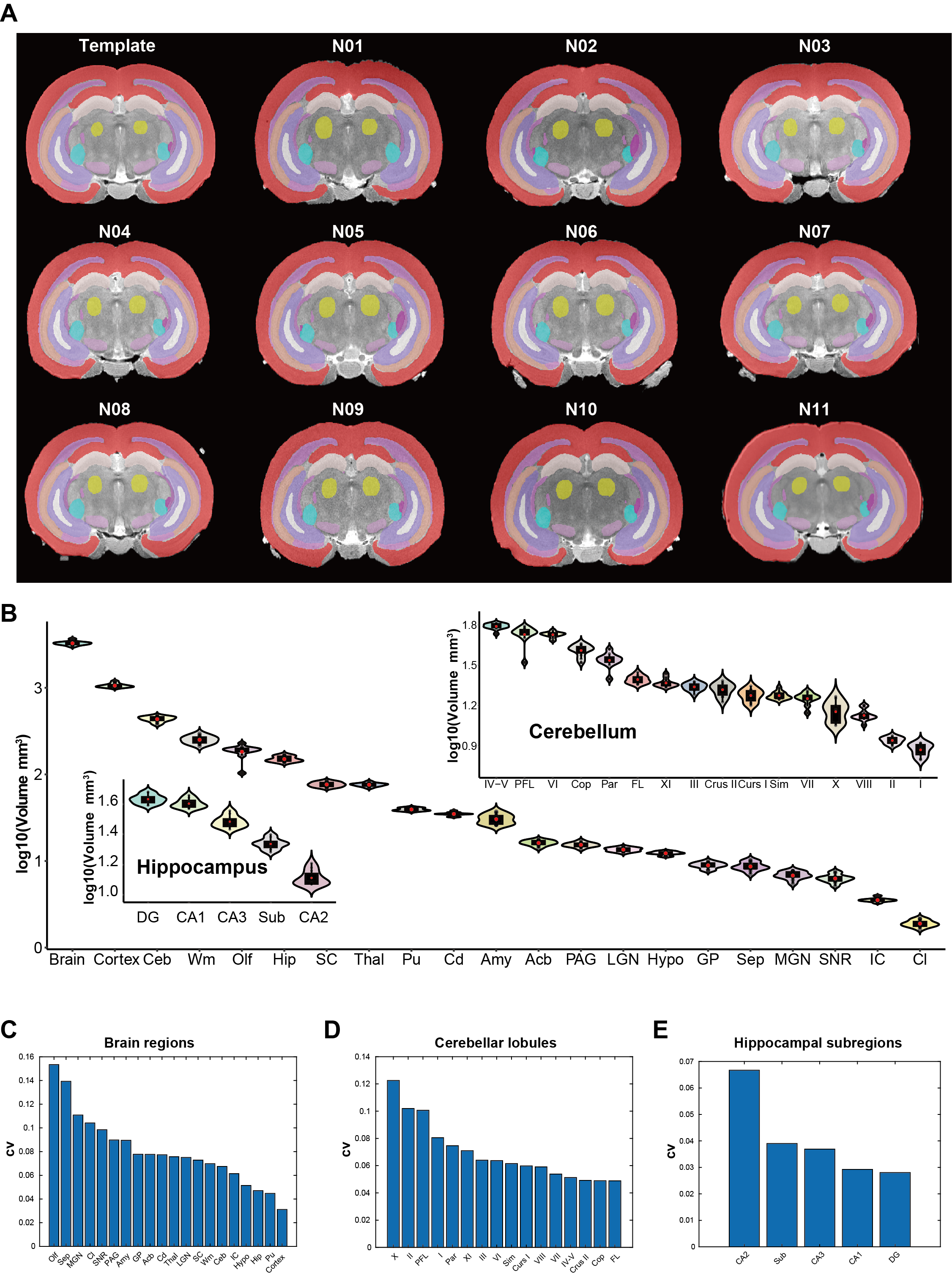

Supplement: S2 Fig — (A) Consistency between template and individual-level parcellations. Comparison of spatial alignment and boundary definitions between the population-averaged template and individual-level brain partitions. (B) Individual-level volumetric distributions of brain regions. Quantitative distribution of brain region volumes across the cohort. Insets highlight the cerebellar lobules (upper right) and hippocampal subdivisions (lower left). Red dots denote population means across all individuals. (C–E) Inter-subject volumetric variability. Coefficient of variation (CV) illustrating the degree of inter-subject variability in individual-level volumes across different anatomical scales: (C) whole-brain partitions, (D) cerebellar lobules, and (E) hippocampal subregions. The data underlying this Figure can be found in S1 Data. (TIF) [file pbio.3003773.s002.tif]

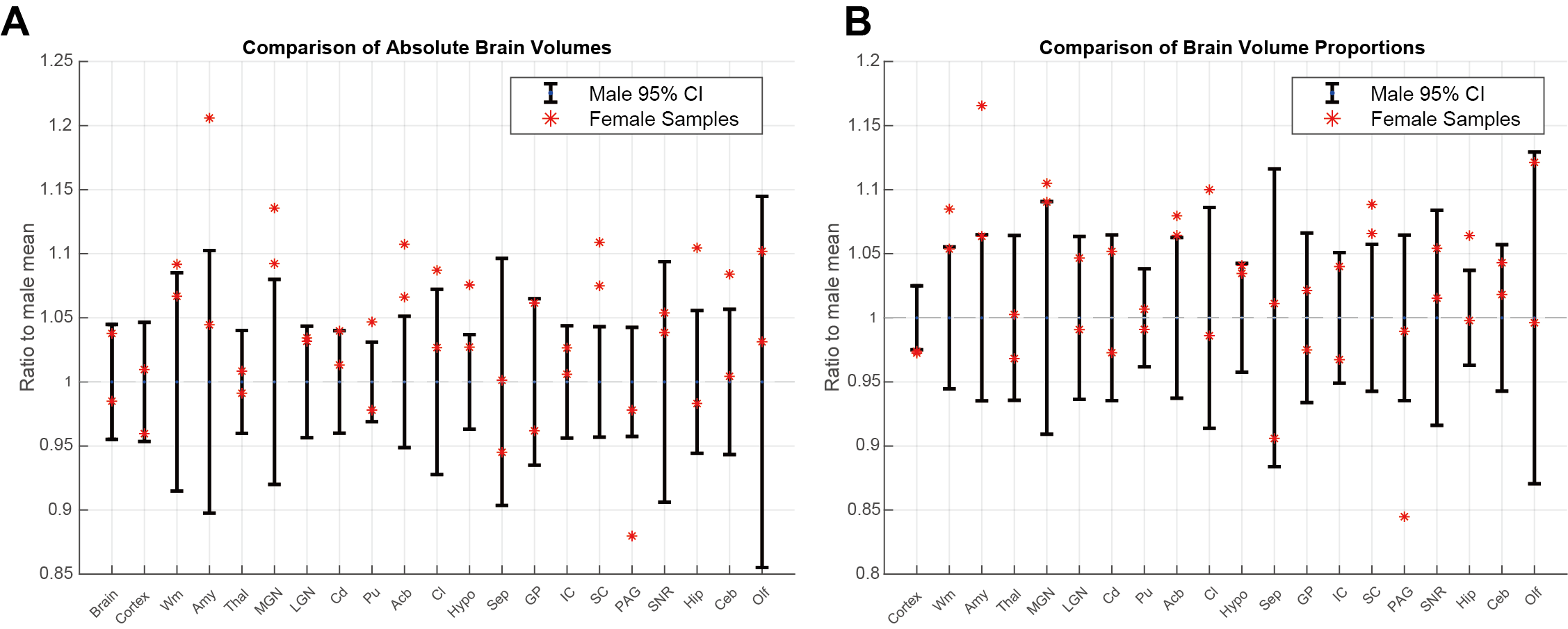

Supplement: S3 Fig — (A) Absolute volume. (B) Relative volume (normalized to total brain volume). The y-axis represents the ratio relative to the male mean for each brain region. Gray horizontal lines (or black error bars) indicate the 95% confidence intervals (CI) for males, while red dots represent the values for females. The data underlying this Figure can be found in S1 Data. (TIF) [file pbio.3003773.s003.tif]

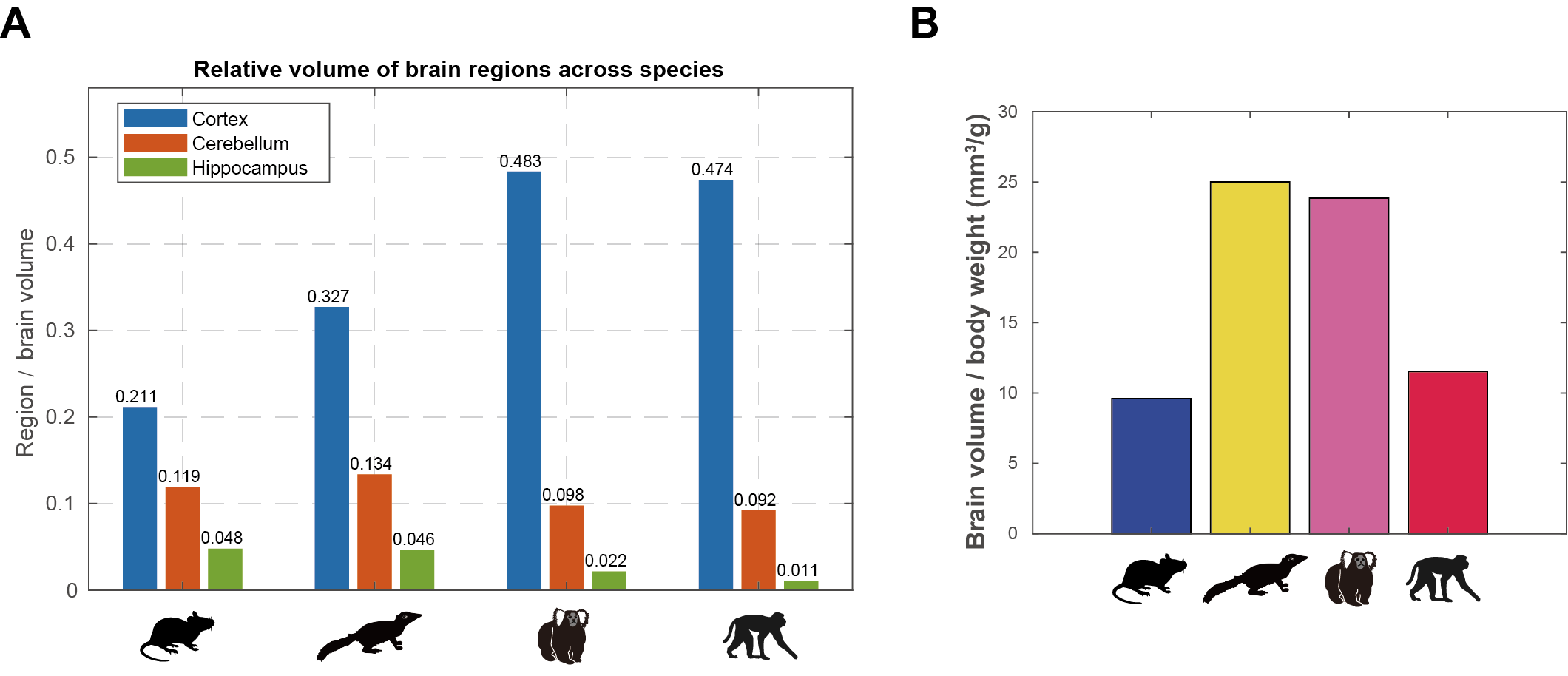

Supplement: S4 Fig — (A) Comparison of relative brain region volumes across species. (B) Absolute brain volume-to-body mass ratios across species, calculated using representative body weights for each species (mouse, 35 g; tree shrew, 130 g; marmoset, 400 g; macaque, 8,000 g). The data underlying this Figure can be found in S1 Data. (TIF) [file pbio.3003773.s004.tif]

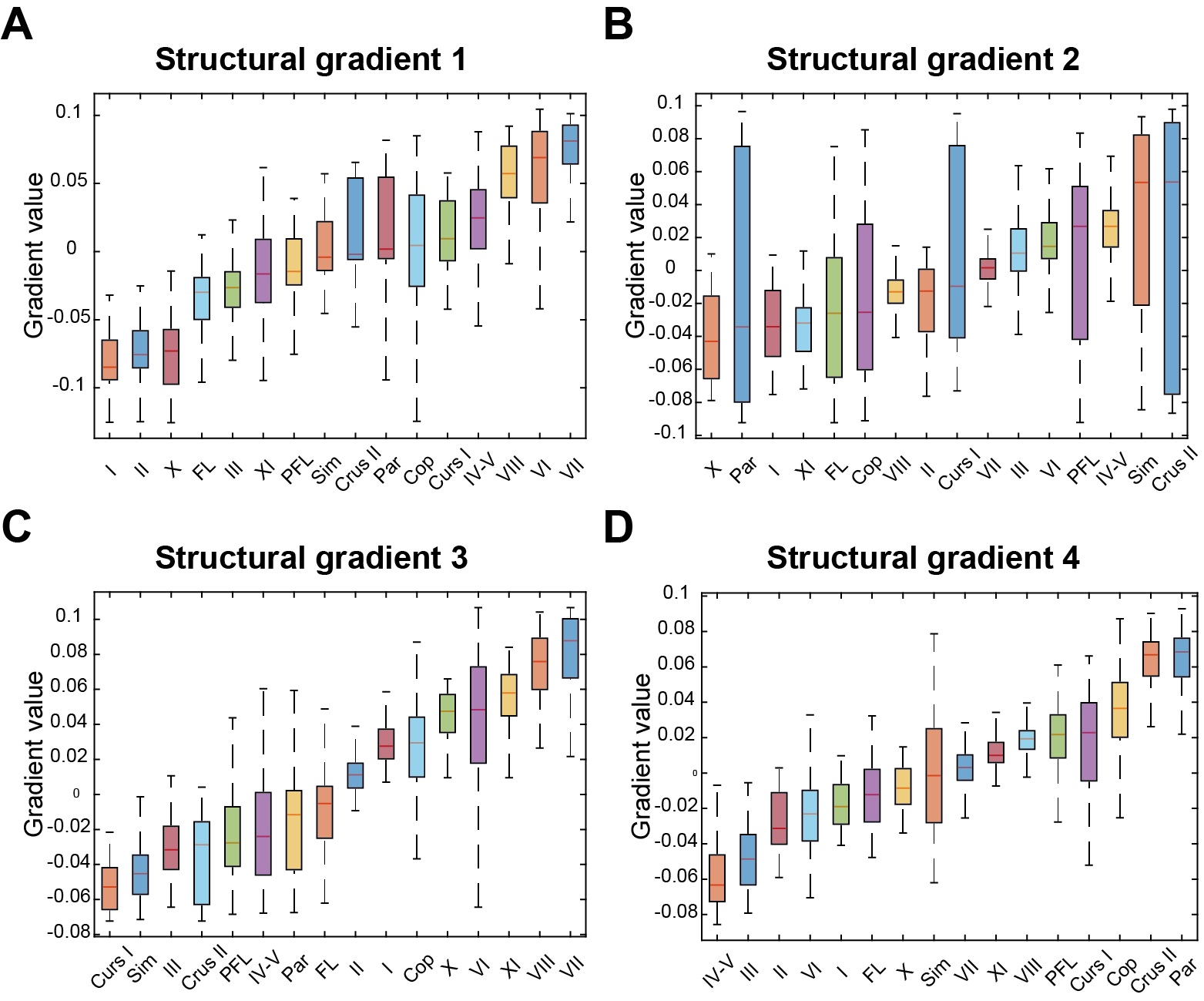

Supplement: S5 Fig — (A–D) Gradient values for gradients 1–4, displayed as a box plot (median and interquartile range, IQR) across cerebellar lobules. The data underlying this Figure can be found in S1 Data. (TIF) [file pbio.3003773.s005.tif]

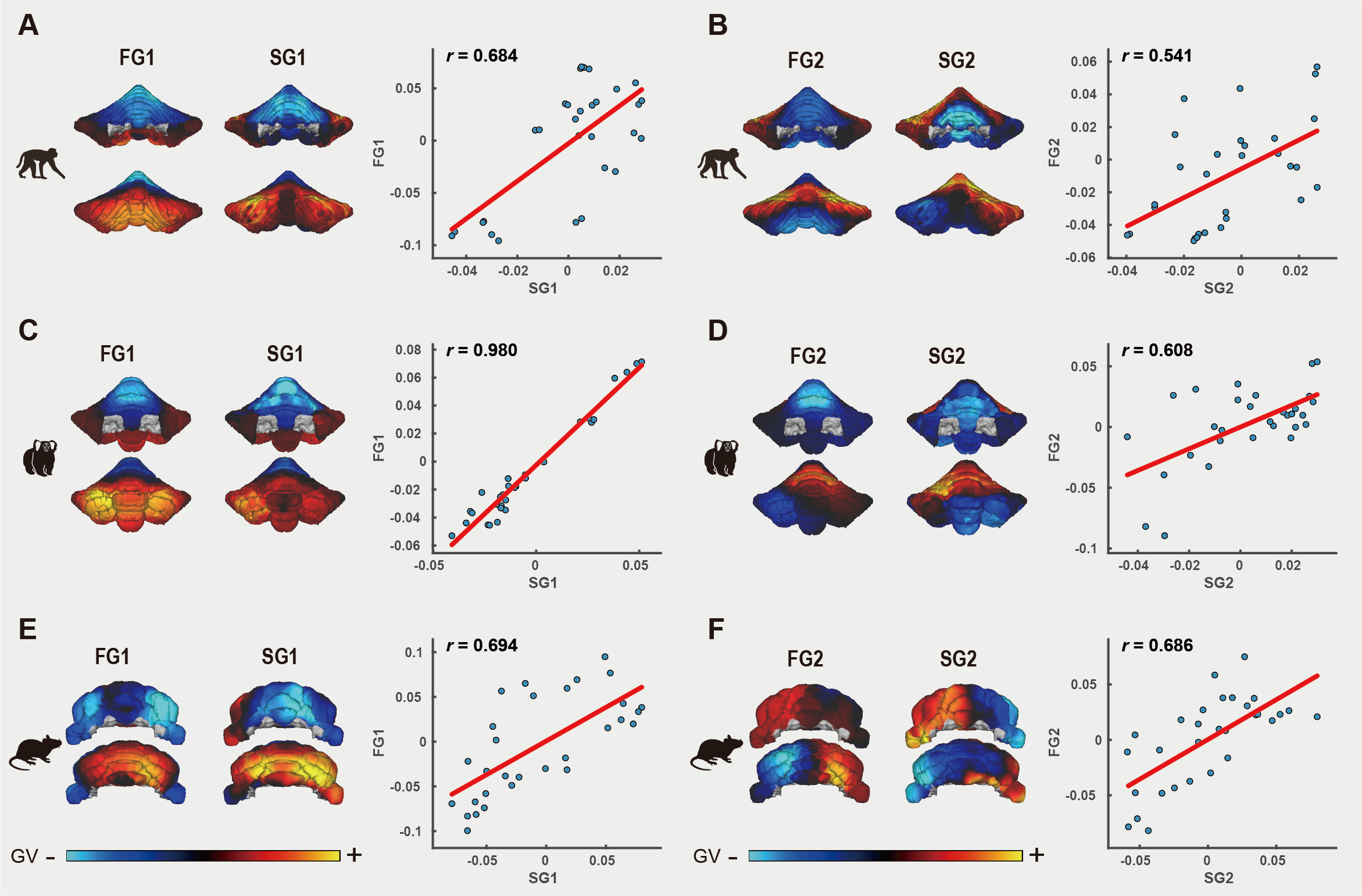

Supplement: S6 Fig — (A–F) The gradients similarity between structural and functional connectivity gradients (SG1–2 and FG1–2) in the macaque (A, B), marmoset (C, D), and mouse (E, F). The similarity was quantified by Pearson correlation coefficients (r) based on the regional-averaged-GV with hemispheric differentiation. The data underlying this Figure can be found in S1 Data. (TIF) [file pbio.3003773.s006.tif]

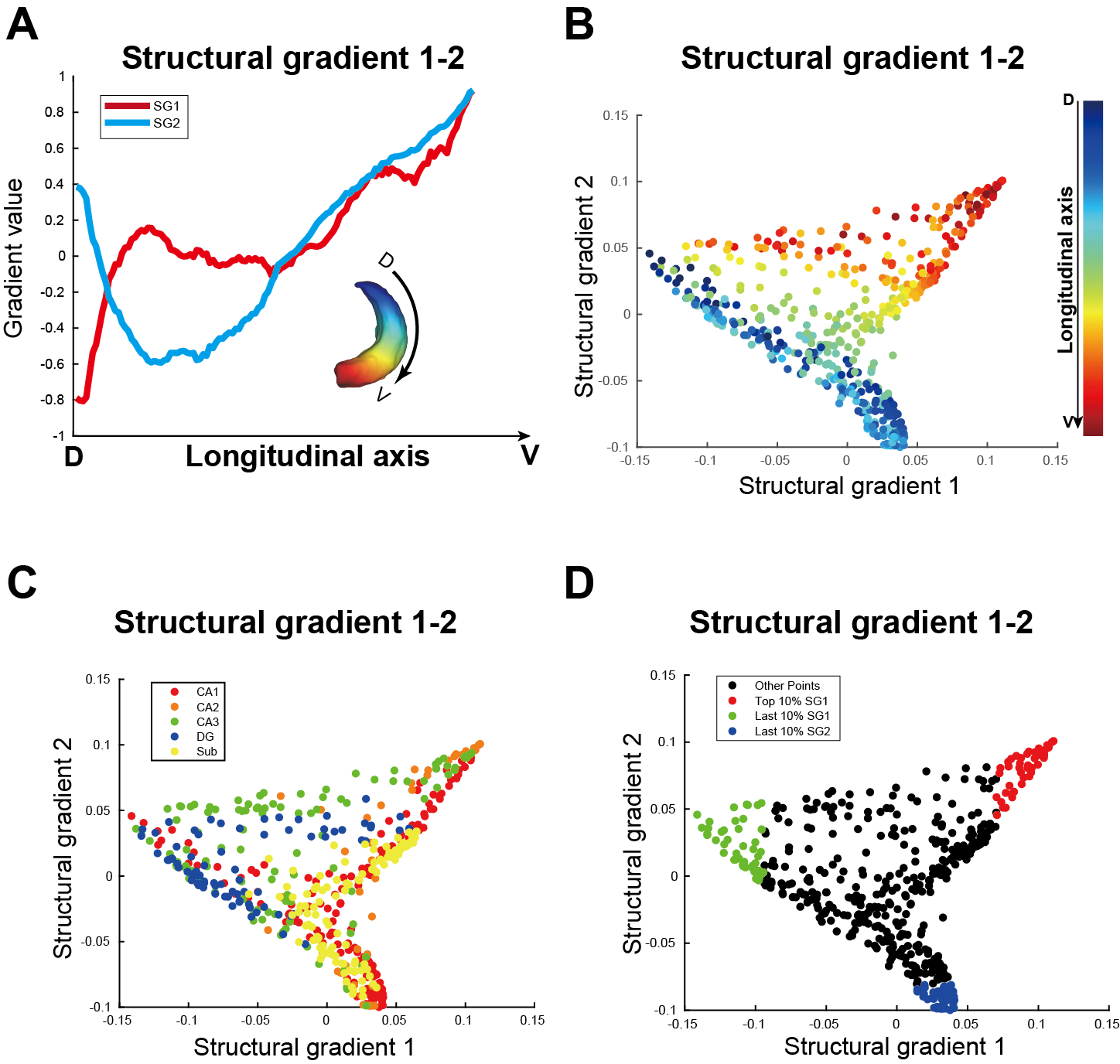

Supplement: S7 Fig — (A–D) Mirroring Fig 5B–E for the right hippocampus. (A) Spatial alignment of SG1 and SG2 values along the longitudinal axis (DV-axis). (B) DV-axis progression of SG1 and SG2 values, color-mapped by DV-axis position. (C) Subregional heterogeneity of SG1 and SG2 across hippocampal subregions (color-coded). (D) Spatial localization of extremal gradient values (top/last 10%) for SG1 and SG2. The data underlying this Figure can be found in S1 Data. (TIF) [file pbio.3003773.s007.tif]

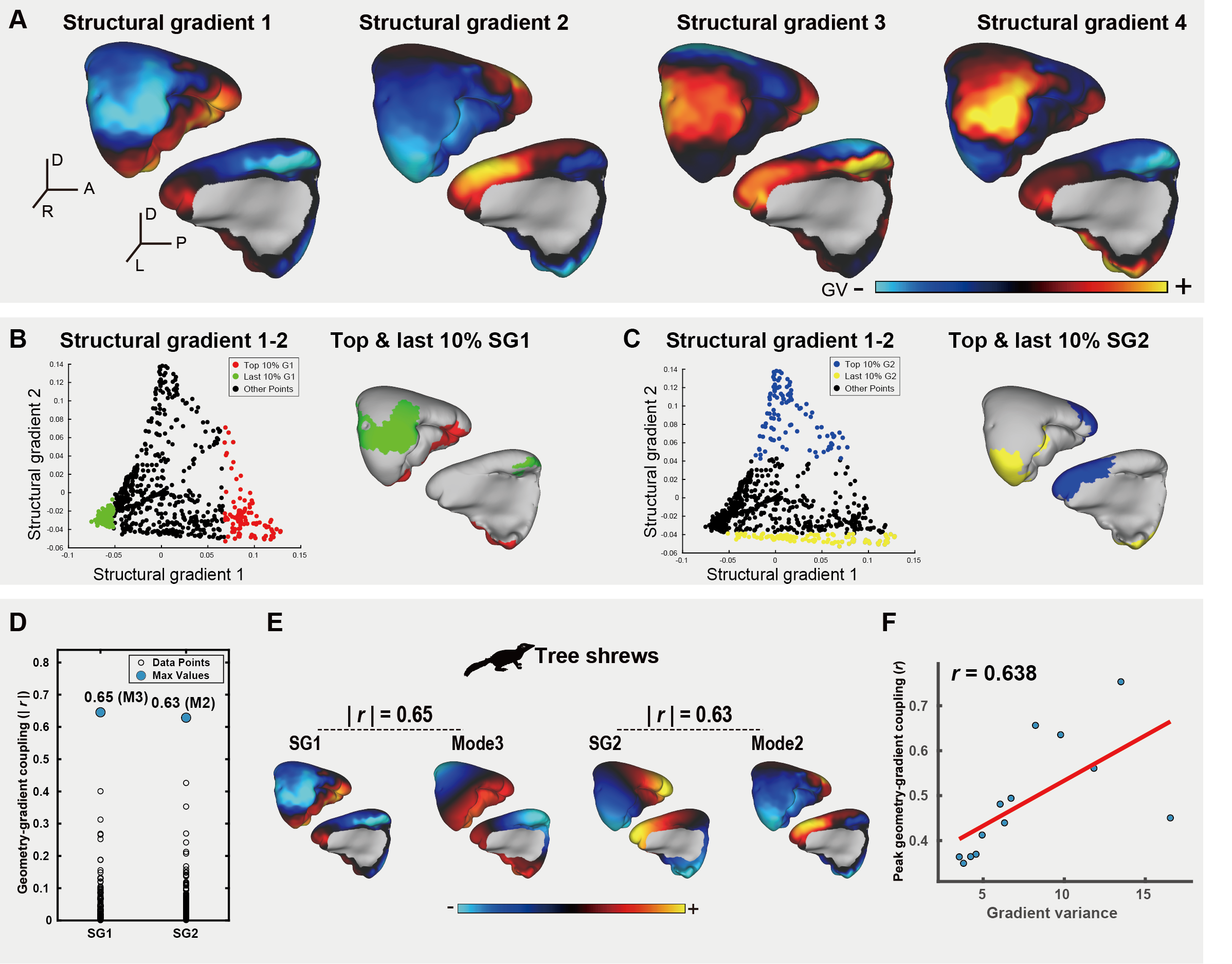

Supplement: S8 Fig — (A–F) Mirroring Fig 6A-F for the right cerebral cortex. (A) Cortical surface maps of the first four structural gradients (SG1–SG4) in the right hemisphere, with color-encoded by gradient value (GV). (B, C) Spatial localization of extremal gradient values (top/last 10%) for SG1 (B) and SG2 (C), overlaid on the surface in the right hemisphere. (D) Correlation spectra quantify absolute Pearson coefficients (| r |) between the first 100 geometric eigenmodes and SG1/SG2 in the tree shrew. Blue circles identify peak GGC values, defined as the maximal coupling strength between each gradient and its optimally correlated geometric eigenmode. (E) Surface mappings of the peak GGC relationships identified in (D) for SG1 (aligned with Mode 3) and SG2 (aligned with Mode 2) in the tree shrew cerebral cortex. (F) Scatterplots demonstrate a robust positive correlation (r = 0.638) between the peak GGC strength (| r | values from D) and gradient-specific explained variance. The data underlying this Figure can be found in S1 Data. (TIF) [file pbio.3003773.s008.tif]

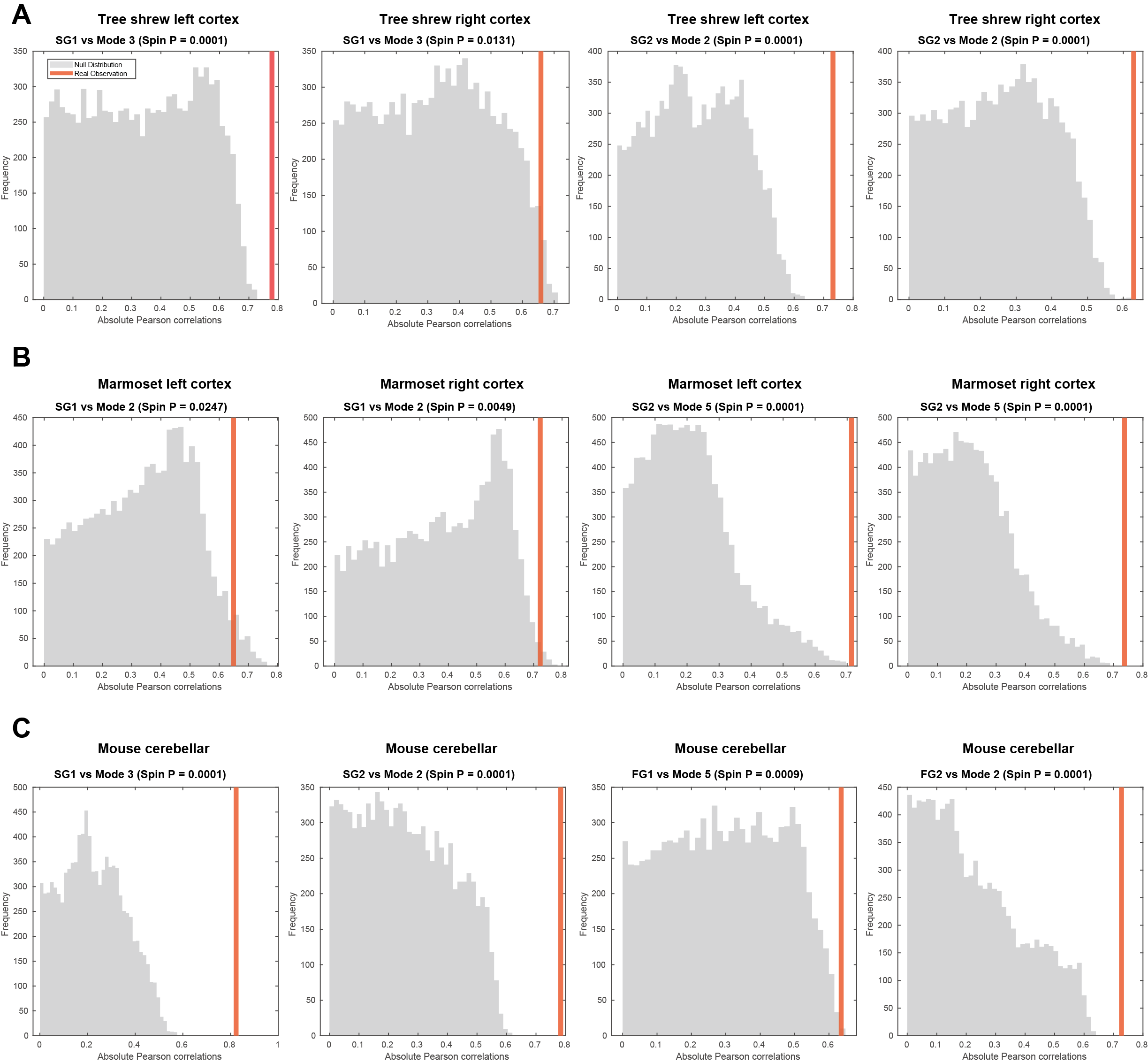

Supplement: S9 Fig — (A, B) Spatial profiles of the first two structural gradients (SG1, SG2) in the left and right cortical hemispheres of the tree shrew (A) and marmoset (B). (C) Structural (SG1–2) and functional (FG1–2) gradients in the mouse cerebellum. Statistical significance of spatial correspondence was assessed using a Spin Test (spatial permutation) preserving spatial autocorrelation. For each hemisphere and species, 10,000 random spherical rotations were applied to remap gradient values while preserving their spatial structure. For each geometric eigenmode, Spearman rank correlation was computed against the 10,000 permuted gradient maps to generate a null distribution. The permutation p-value (Pspin) was calculated as the proportion of null correlations with absolute magnitude ≥ the observed absolute correlation. The data underlying this Figure can be found in S1 Data. (TIF) [file pbio.3003773.s009.tif]

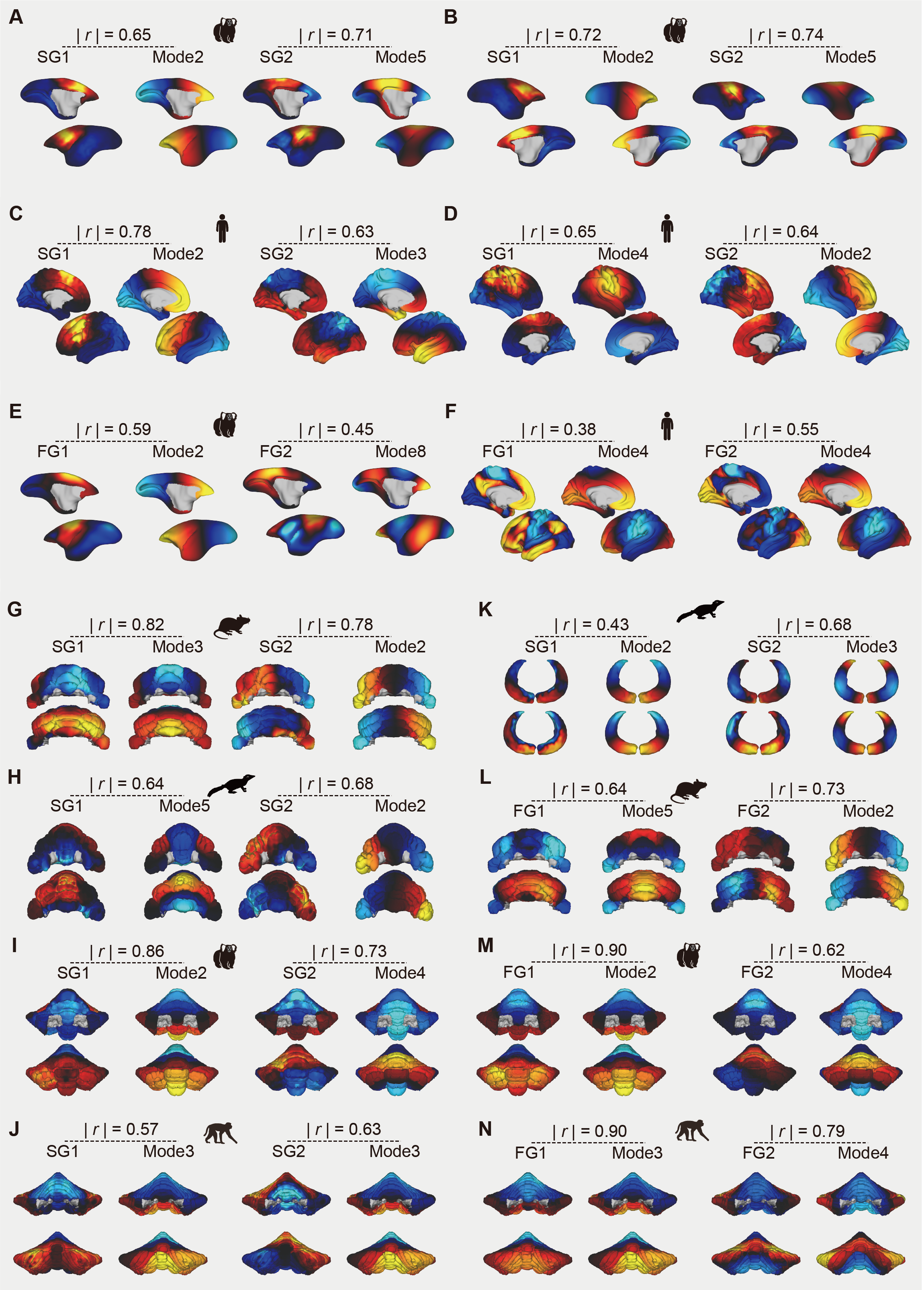

Supplement: S10 Fig — (A–N) Surface mappings of representative peak GGC relationships across domains: (A, B) Structural gradients for the left (A) and right (B) cerebral cortex in the marmoset. (C, D) Structural gradients for the left (C) and right (D) cerebral cortex in human. (E, F) Cortical functional gradients for the left cerebral cortex in the marmoset (E) and human (F). (G–J) Cerebellar structural gradient in mouse (G), tree shrew (H), marmoset (I), and macaque (J). (K) Hippocampal structural gradient (only the GGC values of the left hippocampus was shown). (L–N) Cerebellar functional gradients in the mouse (L), marmoset (M), and macaque (N). Absolute Pearson correlations (| r |) represent peak GGC (maximal coupling strength between region-specific gradients (structural/functional) and their optimally correlated geometric eigenmodes). The data underlying this Figure can be found in S1 Data. (TIF) [file pbio.3003773.s010.tif]

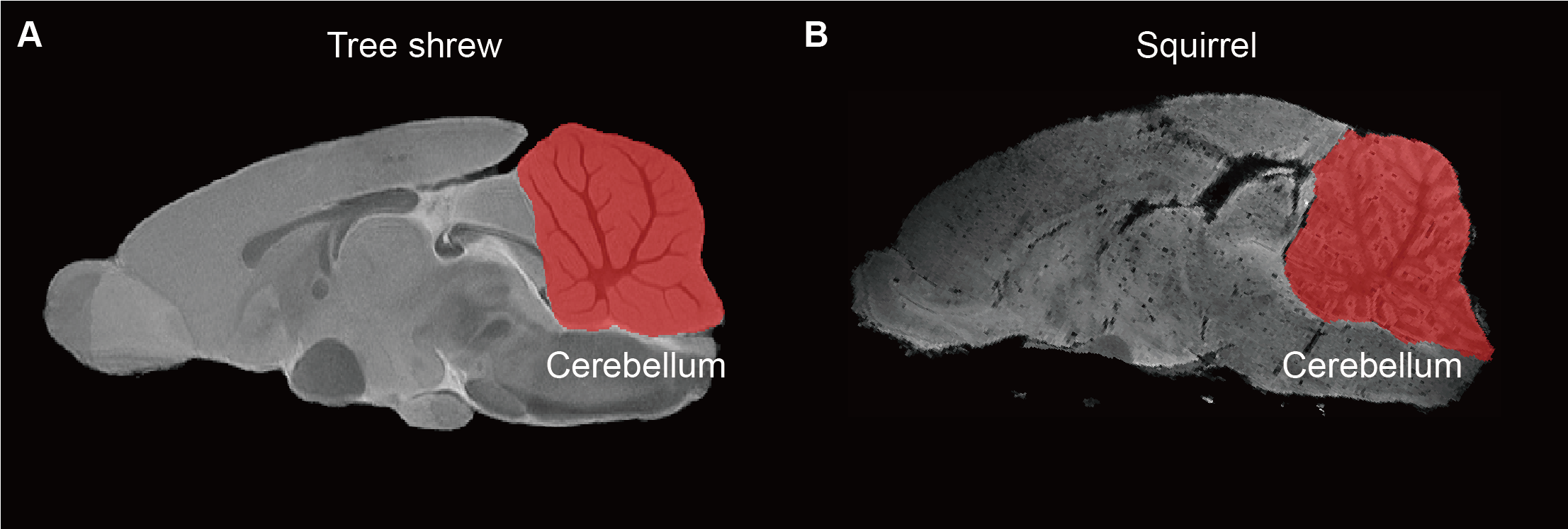

Supplement: S11 Fig — (A) Tree shrew. (B) Red-bellied tree squirrel. The cerebellar regions are highlighted in red, demonstrating the prominent cerebellar volume relative to the whole brain in both arboreal species. The relative cerebellar volume is approximately 13.4% in the tree shrew and 16.3% in the red-bellied tree squirrel. The data for the red-bellied tree squirrel are openly available as part of the tree shrew database at http://www.treeshrewdb.org/MRI/. (TIF) [file pbio.3003773.s011.tif]

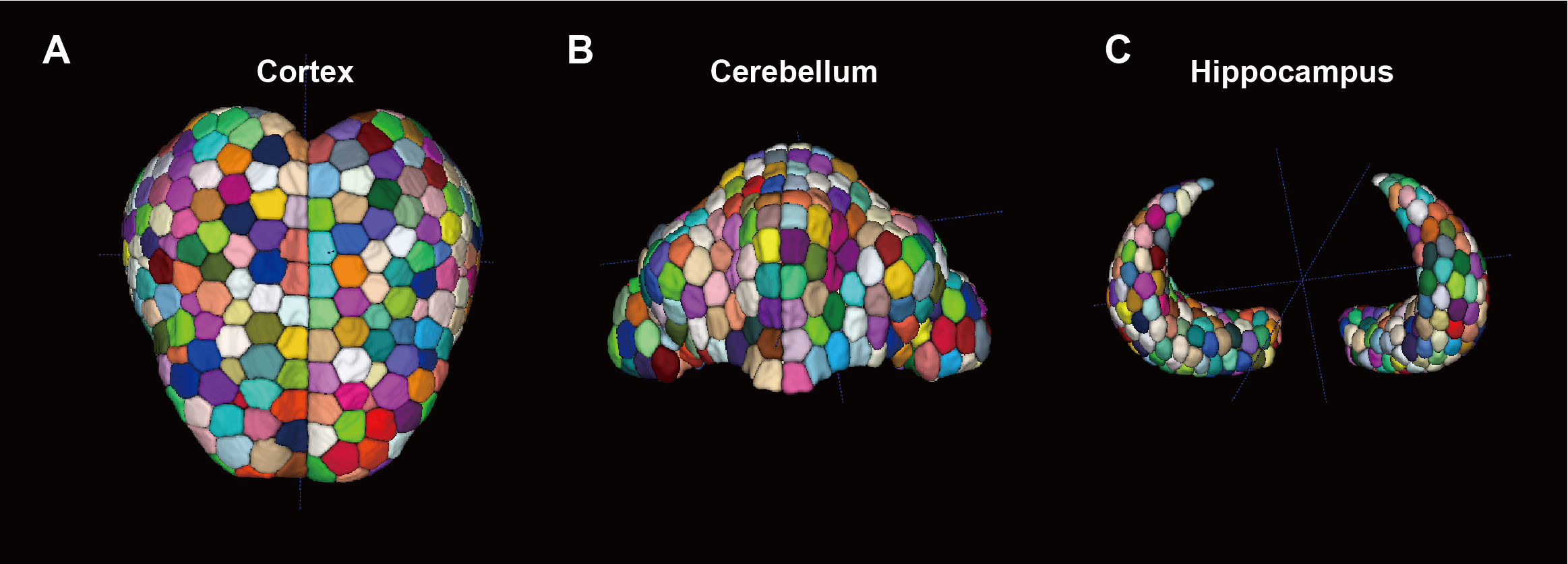

Supplement: S12 Fig — The brain was parcellated into N = 400 distinct regions based on spatial coordinates. Panels illustrate the parcellation results for the (A) cortex, (B) cerebellum, and (C) hippocampus, respectively. This coordinate-based clustering ensures the spatial contiguity and structural integrity of each resulting parcel. (TIF) [file pbio.3003773.s012.tif]

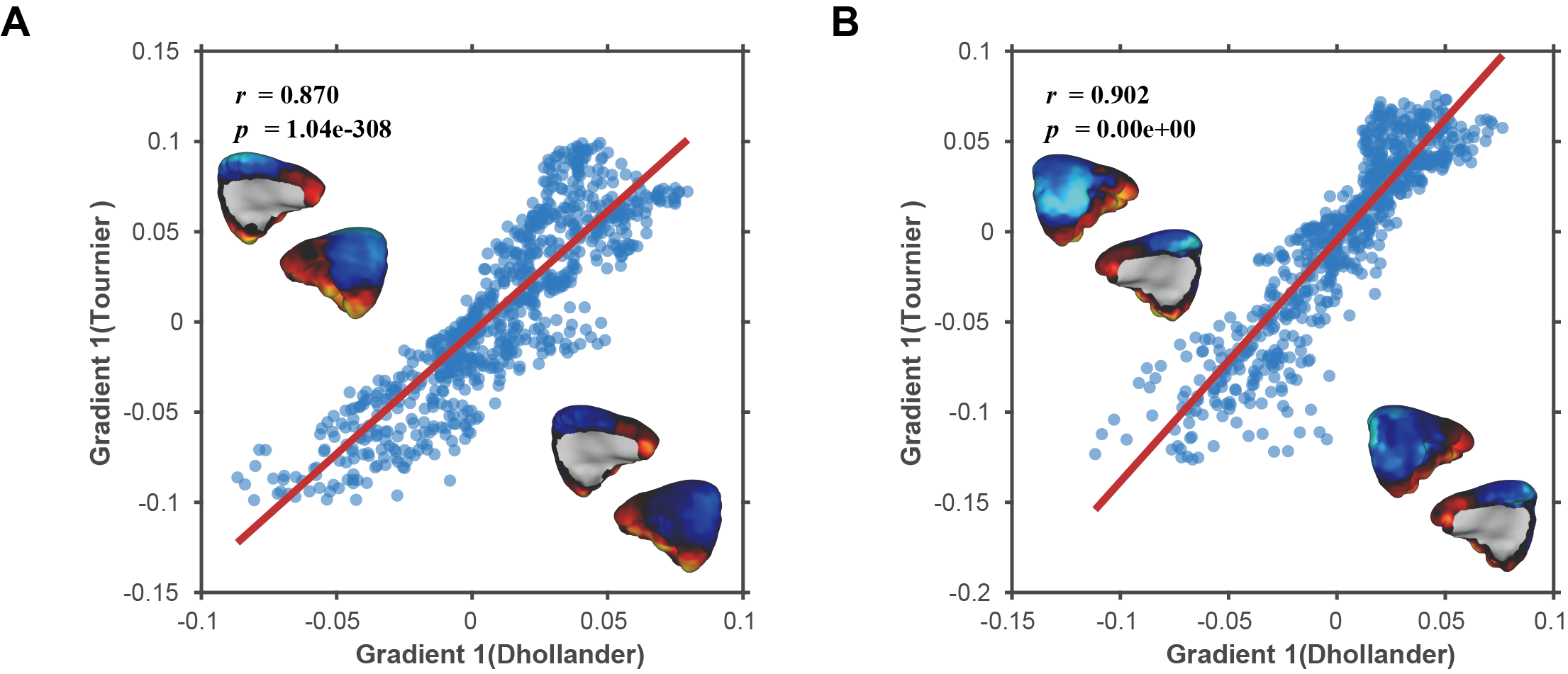

Supplement: S13 Fig — The primary gradients are shown for the left (A) and right hemispheres (B). Spatial similarity between the gradient maps derived from the two algorithms was quantified using Pearson correlation coefficients, where r represents the correlation strength between the two gradient maps and p indicates the statistical significance of this correlation. The high spatial correspondence (left hemisphere: r = 0.90, p < 0.0001; right hemisphere: r = 0.87, p < 0.0001) demonstrates robust estimation across algorithms and consistent bilateral organizational patterns. The data underlying this Figure can be found in S1 Data. (TIF) [file pbio.3003773.s013.tif]

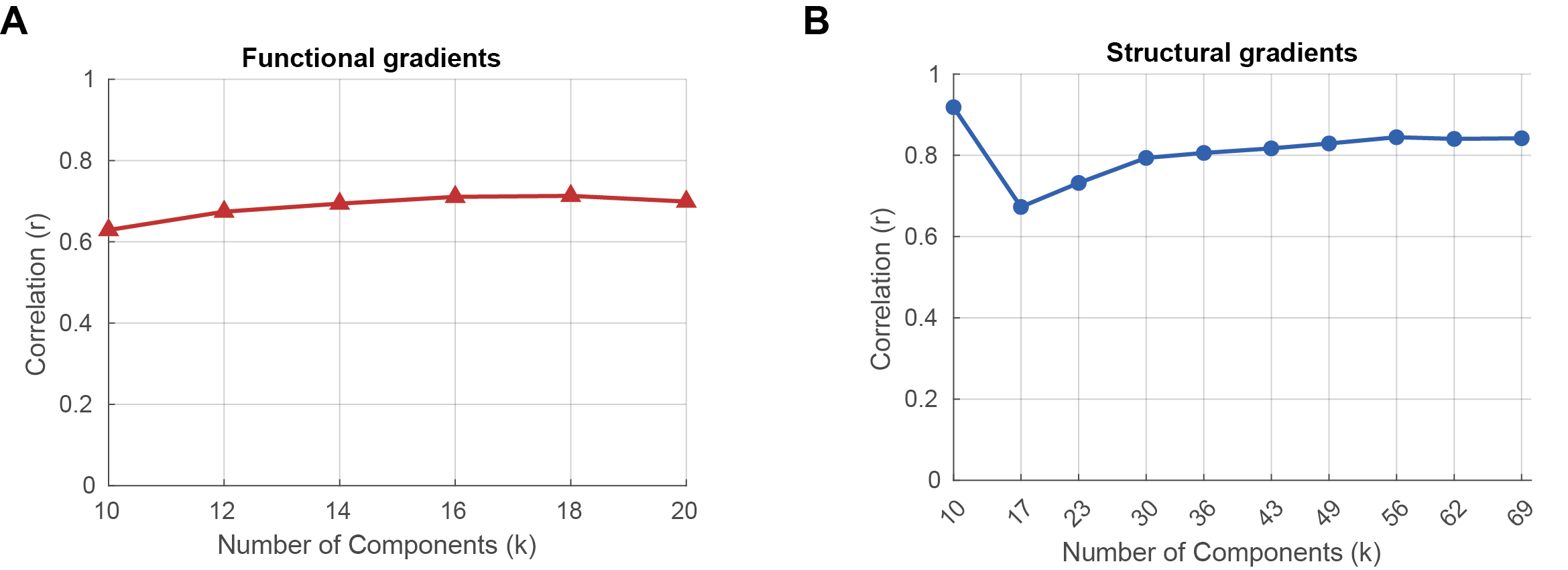

Supplement: S14 Fig — Quantitative validation of the robustness of Geometry-Gradient Correlation (GGC) across varying numbers of gradient components. (A) Functional gradients: The correlation between peak GGC strength and explained variance remains highly stable within the effective range of functional components (k = 10–20). (B) Structural gradients: To test the limits of stability, diffusion MRI data were analyzed across a broader spectrum (k = 10–69). The GGC metric shows remarkable robustness even as higher-order components are included, with no significant degradation or drift in correlation coefficients. The data underlying this Figure can be found in S1 Data. (TIF) [file pbio.3003773.s014.tif]
