# Supplementary material for: An anatomical and connectivity atlas of the tree shrew brain to bridge rodent and primate neuroanatomy
Source: PLoS Biol. 2026 May 4;24(5):e3003773. doi: 10.1371/journal.pbio.3003773 (PMC13138645; doi:10.1371/journal.pbio.3003773)
Supplement: S1 Table — (DOCX) [file pbio.3003773.s015.docx]

**S1 Table. The volume of 20 brain regions.**

| **Regions** | **Brain** | **Cortex** | **Wm** | **Amy** | **Thal** | **MGN** | **LGN** |
| --- | --- | --- | --- | --- | --- | --- | --- |
| **Volume/mm^3^** | 3251.93 | 1063.11 | 248.96 | 31.07 | 75.25 | 6.68 | 13.20 |
| **Regions** | **Cd** | **Pu** | **Acb** | **Cl** | **Hypo** | **Sep** | **GP** |
| **Volume/mm^3^** | 34.65 | 39.43 | 16.26 | 2.04 | 12.52 | 8.87 | 9.04 |
| **Regions** | **IC** | **SC** | **PAG** | **SNR** | **Hip** | **Ceb** | **Olf** |
| **Volume/mm^3^** | 3.59 | 75.81 | 15.25 | 6.47 | 150.64 | 435.19 | 191.47 |

Abbreviations: cerebral cortex (Cortex), cerebral white matter (Wm), amygdala (Amy), thalamus (Thal), medial geniculate nucleus (MGN), lateral geniculate nucleus (LGN), caudate (Cd), putamen (Pu), nucleus accumbens (Acb), claustrum/endopiriform claustrum (Cl), hypothalamus (Hypo), septum (Sep), globus pallidus (GP), inferior colliculus (IC), superior colliculus (SC), periaqueductal gray (PAG), substantia nigra (SNR), hippocampus (Hip), cerebellum (Ceb), and olfactory bulb (Olf).
