# Supplementary material for: An anatomical and connectivity atlas of the tree shrew brain to bridge rodent and primate neuroanatomy
Source: PLoS Biol. 2026 May 4;24(5):e3003773. doi: 10.1371/journal.pbio.3003773 (PMC13138645; doi:10.1371/journal.pbio.3003773)
Supplement: S2 Table — (DOCX) [file pbio.3003773.s016.docx]

**S2 Table. The volume of 16 cerebellar lobules.**

| **Lobules** | **I** | **II** | **III** | **IV-V** | **VI** | **VII** | **VIII** | **IX** |
| --- | --- | --- | --- | --- | --- | --- | --- | --- |
| **Volume/mm^3^** | 7.40 | 8.80 | 21.84 | 61.47 | 53.64 | 17.80 | 13.34 | 23.10 |
| **Lobules** | **X** | **FL** | **Cop** | **Par** | **Sim** | **Crus I** | **Crus II** | **PFL** |
| **Volume/mm^3^** | 14.10 | 24.89 | 40.48 | 33.94 | 19.35 | 18.92 | 20.70 | 55.45 |

Abbreviation: lingula I (I), central lobule II (II), culmen III (III), declive IV (IV), lobule V (V), folium VI (VI), tuber VII (VII), pyramid VIII (VIII), uvula IX (IX), nodulus X (X), simplex lobule (SIM), paramedian lobule (Par), copula (Cop), ﬂocculus (Fl), paraﬂocculus (PFI), Crus I, and Crus II.
