# Supplementary material for: An anatomical and connectivity atlas of the tree shrew brain to bridge rodent and primate neuroanatomy
Source: PLoS Biol. 2026 May 4;24(5):e3003773. doi: 10.1371/journal.pbio.3003773 (PMC13138645; doi:10.1371/journal.pbio.3003773)
Supplement: S3 Table — (DOCX) [file pbio.3003773.s017.docx]

**S3 Table. The volume of 5 hippocampal subregions.**

| **Subregions** | **CA1** | **CA2** | **CA3** | **DG** | **Sub** |
| --- | --- | --- | --- | --- | --- |
| **Volume/mm^3^** | 41.24 | 13.03 | 30.62 | 43.18 | 22.56 |

Abbreviations: cornu ammonis1 (CA1), cornu ammonis2 (CA2), cornu ammonis3 (CA3), dentate gyrus (DG), and subiculum (Sub).
