# Supplementary material for: An anatomical and connectivity atlas of the tree shrew brain to bridge rodent and primate neuroanatomy
Source: PLoS Biol. 2026 May 4;24(5):e3003773. doi: 10.1371/journal.pbio.3003773 (PMC13138645; doi:10.1371/journal.pbio.3003773)
Supplement: S4 Table — (DOCX) [file pbio.3003773.s018.docx]

**S4 Table.** **The surface area of 16 cerebellar lobules.**

| **Lobules** | **I** | **II** | **III** | **IV-V** | **VI** | **VII** | **VIII** | **IX** |
| --- | --- | --- | --- | --- | --- | --- | --- | --- |
| **Area/mm^2^** | 12.76 | 17.15 | 42.80 | 135.69 | 81.72 | 32.77 | 28.98 | 51.04 |
| **Lobules** | **X** | **FL** | **Cop** | **Par** | **Sim** | **Crus I** | **Crus II** | **PFL** |
| **Area/mm^2^** | 19.53 | 50.85 | 72.34 | 75.75 | 54.73 | 44.77 | 42.28 | 141.94 |
