# Supplementary material for: An anatomical and connectivity atlas of the tree shrew brain to bridge rodent and primate neuroanatomy
Source: PLoS Biol. 2026 May 4;24(5):e3003773. doi: 10.1371/journal.pbio.3003773 (PMC13138645; doi:10.1371/journal.pbio.3003773)
Supplement: S5 Table — (DOCX) [file pbio.3003773.s019.docx]

**S5 Table.** **Summary of regional brain metrics across species.**

|  | Mouse | Tree shrew | Marmoset | Macaque |
| --- | --- | --- | --- | --- |
| Brain volume (mm³) | 336.0211 | 3251.9310 | 9540.4104 | 92238.4531 |
| Cortical volume (mm³) | 71.0675 | 1063.1134 | 4612.3244 | 43694.8125 |
| Cerebellar volume (mm³) | 39.9342 | 435.2198 | 931.2108 | 8488.2500 |
| Hippocampal volume (mm³) | 16.1178 | 150.6375 | 205.8281 | 997.9531 |
| Cortical / Brain volume | 0.2115 | 0.3269 | 0.4835 | 0.4737 |
| Cerebellar / Brain volume | 0.1188 | 0.1338 | 0.0976 | 0.0920 |
| Hippocampal / Brain volume | 0.0480 | 0.0463 | 0.0216 | 0.0108 |
| Brain volume / Body weight (mm³/g) | 9.6006 | 25.0149 | 23.8510 | 11.5298 |
| Cortical surface areas (mm²) | 152.2049 | 1229.4800 | 2898.8601 | 20872.4410 |
| Cerebellar pial surface areas (mm²) | 134.1659 | 905.0960 | 1782.8072 | 7781.7562 |
| Cerebellar contour surface areas (mm²) | 79.1955 | 394.6665 | 611.1296 | 2443.5000 |
| Cerebellar-to-neocortical surface area ratio | 0.8815 | 0.7362 | 0.6150 | 0.3728 |
| Cerebellar gyrification index | 1.6941 | 2.2933 | 2.9172 | 3.1847 |
